# Regulation of Ion Channel Function in Human-Induced Pluripotent Stem Cell-Derived Cardiomyocytes by Cancer Cell Secretion Through DNA Methylation

**DOI:** 10.3389/fcvm.2022.839104

**Published:** 2022-02-21

**Authors:** Rujia Zhong, Feng Zhang, Zhen Yang, Yingrui Li, Qiang Xu, Huan Lan, Siegfried Lang, Lukas Cyganek, Elke Burgermeister, Ibrahim El-Battrawy, Xiaobo Zhou, Ibrahim Akin, Martin Borggrefe

**Affiliations:** ^1^First Department of Medicine, Medical Faculty Mannheim, University Medical Centre Mannheim (UMM), University of Heidelberg, Mannheim, Germany; ^2^Key Laboratory of Medical Electrophysiology of Ministry of Education and Medical Electrophysiological Key Laboratory of Sichuan Province, Institute of Cardiovascular Research, Southwest Medical University, Luzhou, China; ^3^DZHK (German Center for Cardiovascular Research), Partner Site, Mannheim, Germany; ^4^Stem Cell Unit, Clinic for Cardiology and Pneumology, University Medical Center Göttingen, Göttingen, Germany; ^5^DZHK (German Center for Cardiovascular Research), Partner Site, Göttingen, Germany; ^6^Second Department of Medicine, Faculty of Medicine, University Medical Centre Mannheim (UMM), University of Heidelberg, Mannheim, Germany

**Keywords:** cancer cell secretion, human-induced pluripotent stem cell-derived cardiomyocyte, ion channel, DNA methylation, arrhythmia

## Abstract

**Background:**

Cardiac dysfunction including arrhythmias appear frequently in patients with cancers, which are expected to be caused mainly by cardiotoxic effects of chemotherapy. Experimental studies investigating the effects of cancer cell secretion without chemotherapy on ion channel function in human cardiomyocytes are still lacking.

**Methods:**

The human-induced pluripotent stem cell-derived cardiomyocytes (hiPSC-CMs) generated from three healthy donors were treated with gastrointestinal (GI) cancer (AGS and SW480 cells) medium for 48 h. The qPCR, patch-clamp, western blotting, immunostaining, dot blotting, bisulfite sequence, and overexpression of the ten-eleven translocation (TET) enzyme were performed for the study.

**Results:**

After treated with cancer cell secretion, the maximum depolarization velocity and the action potential amplitude were reduced, the action potential duration prolonged, peak Na^+^ current, and the transient outward current were decreased, late Na^+^ and the slowly activating delayed rectifier K^+^ current were increased. Changes of mRNA and protein level of respective channels were detected along with altered DNA methylation level in CpG island in the promoter regions of ion channel genes and increased protein levels of DNA methyltransferases. Phosphoinositide 3-kinase (PI3K) inhibitor attenuated and transforming growth factor-β (TGF-β) mimicked the effects of cancer cell secretion.

**Conclusions:**

GI cancer cell secretion could induce ion channel dysfunction, which may contribute to occurrence of arrhythmias in cancer patients. The ion channel dysfunction could result from DNA methylation of ion channel genes *via* activation of TGF-β/PI3K signaling. This study may provide new insights into pathogenesis of arrhythmia in cancer patients.

## Background

Cardiovascular disease (CVD) and cancer are the two most common causes of mortality worldwide (1). Data from World Health Organization in 2018 show that about 18 million people die from CVD annually worldwide (2), while about 18 million patients suffer from and about 10 million die of cancers (3). Improved diagnostic and therapeutic modalities achieve remarkable progress in improving the survival rates of cancer patients. However, the rise in the incidence of CVD among cancer patients has drawn attention. It has been reported that the incidence of CVD is 10-fold higher in cancer survivors than that in controls, along with higher rates of congestive heart failure and stroke, and higher incidence of cardiovascular risk factors (4, 5). Even worse, in some cancer patients, cardiovascular deaths are more common than cancer deaths (6). Cardiotoxicity of anti-cancer therapies is a common anti-cancer therapy related adverse effect, but cancer itself could affect cardiovascular system. Evidence has revealed that tumors can cause arrhythmia in cancer patients through direct infiltration, systematic inflammation, metabolic derangements, impaired oxygenation, and electrolyte or endocrine abnormalities (7). A study demonstrated that heart rate variability and cardiorespiratory coordination were changed in patients with breast cancer (8). Another study on acute leukemia patients found that cardiac autonomic functions were changed with a decrease in heart rate variability (9). These findings further demonstrate that, apart from anti-cancer toxicity, a tumor may itself have a deleterious effect on myocardial function of the heart. A study in mouse model also reported that mice injected with lung carcinoma cells showed degenerative structural cardiac lesions and a significant reduction in myocardial innervation (10). Overall, the aforementioned evidence indicates that cancer may affect the electrophysiology of cardiomyocytes, independent of cancer therapy.

Gastrointestinal (GI) cancers are a group of highly aggressive malignancies with heavy cancer-related mortalities (11). It has been reported that patients with GI cancers showed lower heart rate variability (12). A study found that patients with colorectal cancer have a 2-fold higher incidence of atrial fibrillation than patients with non-neoplastic diseases (13). However, there are no experimental studies investigating the potential role of GI cancers on cardio electrophysiology and the underlying mechanisms.

In the present study, we aim to assess the possible cardiac effects induced by GI cancer cell secretion and explore their possible mechanisms using human-induced pluripotent stem cell-derived cardiomyocytes (hiPSC-CMs).

## Methods

### Ethics Statements

The skin biopsy from three healthy donors was obtained with written informed consent. The generation and application of hiPSC-CMs have been approved by the Ethics Committee of Medical Faculty Mannheim, Heidelberg University (approval number: 2018-565N-MA) and by the Ethics Committee of the University Medical Center Göttingen (approval number: 10/9/15). This study was conducted in accordance with the ethical principles that have their origin in the Declaration of Helsinki of 1975.

### Cell Culture and Differentiation of HiPSCs

The human-induced pluripotent stem cells (hiPSCs) were generated from primary fibroblasts derived from skin biopsies of three healthy donors. The generation of hiPSC-CMs from hiPSCs have been described in our previous studies (14, 15). The successfully differentiation of hiPSC-CMs was confirmed by immunofluorescence of cardiac markers α-actinin and myosin light chain 4 (Myl4) (Supplementary Figure 1) together with spontaneous cell beating and cardiac action potential features.

The two cancer cell lines AGS (ATCC® CRL-1739™) and SW480[SW-480] (ATCC® CCL-228™) were bought from ATCC (LGC Standards GmbH, Wesel, Germany). Cancer cells (1 × 10^5^ cells) were seeded in T75 flasks, and then cultured in DMEM (10 mL) supplemented with 10% FBS and 1% Pen/Strep. According to the growth curve of the cell lines by cell counting and MTT assay (Supplementary Figure 2), we harvested the cancer cell media on day 8 at the end of the logarithmic growth phase with stable cancer cell number and secretion. PCR data showed that different concentrations of the fresh cancer cell media had no relevant effects on the ion channel gene expressions of hiPSC-CMs (Supplementary Figure 3). Since the medium cultured with cancer cells showed concentration-dependent effects (Supplementary Figure 4), we chose the concentration of 20% of cancer cell media for the further experiments.

### Patch-Clamp

All the experiments of action potentials (APs) and ion channel currents were measured by whole-cell patch clamp recording techniques with ISO-3 patch-clamp software (MFK M. Friedrich, Niedernhausen, Germany) and carried out at room temperature (22–25°C). APs were provoked by pulse stimulations (1nA for 5 ms) at 1, 2, and 3 Hz. The channel currents were analyzed with different protocols, solutions and channel blockers. Solutions for Patch-clamp experiments were listed in Supplementary Methods.

### Western Blotting

The protein samples were loaded 20μg per sample for SDS-PAGE, and then transferred to the PVDF membrane (IPVH00010, Merck KGaA, Darmstadt, Germany). After blocking with 5% non-fat milk for 1 h at room temperature, the membranes were incubated with the primary antibodies (shown in Supplementary Methods) at 4°C overnight and then secondary antibodies (shown in Supplementary Methods). The target protein bands were quantified by Fusion Solo system (Vilber, France) and measured with software (Image J software, Research Services Branch, National Institute of Mental Health, Bethesda, MD, USA) for statistical analyses.

### Dot Blotting

The genome DNA, extracted from hiPSC-CMs by using phenol-chloroform extraction, was serially diluted and denatured in 0.1 M NaOH/10 mM EDTA at 95°C for 10 min. The denatured DNA was spotted on 0.2 μm nitrocellulose membrane and air-dry. The membrane was washed with 20 × SSC buffer (175.3 g NaCl, 88.2 g trisodium citrate, pH 7.0 in 1 L ddH_2_O) and then soaked in denaturation solution (1.5 M NaCl, 0.5 M NaOH) for 10 min, then in neutralization solution (1 M NaCl, 0.5 M Tris-HCl pH 7.0) for 5 min. Then the membrane was baked for 2 h at 80°C. After blocking the membrane with 5% non-fat milk in TBST for 1 h at room temperature, the membrane was incubated with anti-5-methylcytidine antibody (ab10805, Abcam, UK) and recombinant anti-5-hydroxymethylcytosine antibody [RM236] (ab214728, Abcam, UK) at 4°C overnight. Then the membrane was incubated with secondary antibodies for 1 h at room temperature. The membrane was imaged by Fusion Solo system (Vilber, France).

### Bisulfite Sequence and Polymerase Chain Reaction

The bisulfite conversion of genome DNA from different groups was performed with EpiTect Bisulfite Kits (59104, QIAGEN, Germany). The promoter sequences of ion channel genes were obtained from UCSC Genome Browser (https://genome.ucsc.edu/). Bisulfite sequence PCR primers for CpG island of ion channel genes were designed by using Methyl Primer Express v1.0 software (Thermo Fisher Scientific, USA) and shown in Supplementary Table 1. PCR amplification was performed using TaKaRa EpiTaq HS (for bisulfite-treated DNA) kit (R110A, Takara, Japan). The PCR products were loaded for agarose gel electrophoresis and purified using Wizard SV Gel and PCR Clean-Up System (A9281/2/5, Promega, USA). The purified PCR products were inserted into pMD20-T vector (purchased from TAKARA, Cat. No. 9128) with TaKaRa Mighty TA-cloning Kit (6028, Takara, Shiga, Japan) and transformed to *E. coli* HST08 Premium Competent Cells (9128, Takara, Shiga, Japan). The plasmids were amplified in the E. coli cells and then extracted with Quick Plasmid Miniprep Kit (K210010, Thermo Fisher Scientific, USA) for sequencing (Eurofins Genomics Germany GmbH, Ebersberg, Germany).

### Overexpression of the Ten-Eleven Translocation 1 Enzyme

The overexpression of Ten-Eleven Translocation 1 Enzyme (TET1) gene was accomplished by transfection of TET1 plasmid FH-TET1-pEF. An empty backbone plasmid pEF1a-IRES-Neo was used as the empty control. FH-TET1-pEF was a gift from Anjana Rao (Addgene plasmid # 49792; http://n2t.net/addgene:49792; RRID:Addgene_49792) and pEF1a-IRES-Neo was a gift from Thomas Zwaka (Addgene plasmid # 28019; http://n2t.net/addgene:28019; RRID:Addgene_28019). The transfection was performed by using FuGENE® HD Transfection Reagent (E2311, Promega, WI, USA) according to the protocol provided by Promega.

### Transforming Growth Factor-β Treatment of Cardiomyocytes

To confirm the effect of transforming growth factor-β (TGF-β) on the ion channel expression, we treated the cardiomyocytes with TGF-β (ab50036, Abcam, UK) at 60 pg/ml for 48 h.

### Statistics

All statistical data were expressed as mean ± standard error of the mean (SEM). Statistical analyses were performed by GraphPad Prism 7 (GraphPad Software Inc., California, USA). Student's *t*-test, one-way analysis of variance (ANOVA), and two-way ANOVA with Tukey's multiple comparisons post-test were applied. *P* < 0.05 were considered statistically significant. The *n*-values in each figure legend reflected the number of cells used for the statistical analysis.

## Results

### Effects of Cancer Cell Secretion on Action Potentials and Ion Channels of HiPSC-CMs

**Figure 1:**
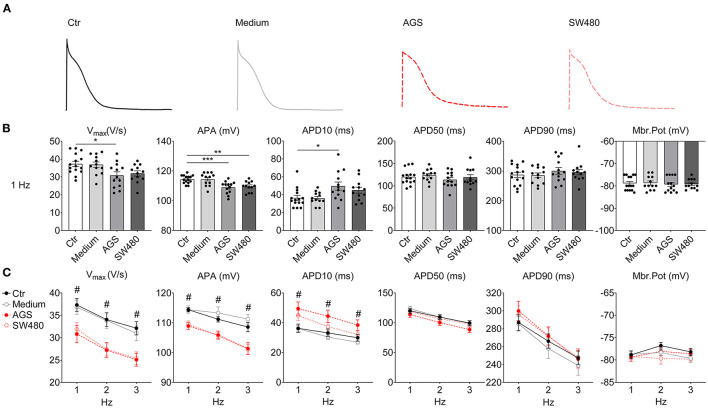
Action potential (AP) characteristics of hiPSC-CMs cultured with AGS and SW480 cancer cell media. APs were recorded by patch clamp (whole cell configuration) measurements in hiPSC-CMs pretreated with culture medium from flask containing cancer cells or no cancer cells. **(A)** Examples of original AP traces of 1 Hz in each group. **(B)** Mean values of AP parameters including the AP amplitude (APA), maximum depolarization velocity (V_max_), AP duration at 10% (APD10), 50% (APD50), and 90% (APD90) repolarization and resting potential (RP). **(C)** Mean values of AP parameters at 1, 2, and 3 Hz. “Ctr” represents data from hiPSC-CMs without medium of cancer cells. “Medium” represents data from hiPSC-CMs with addition of fresh medium for cancer cells. “AGS” represents data from hiPSC-CMs with addition of cultured medium of AGS cancer cells. “SW480” represents data from hiPSC-CMs with addition of cultured medium of SW480 cancer cells. Data are presented as mean ± SEM and analyzed by one-way ANOVA. Cell numbers: *n* = 15 in Ctr, *n* = 12 in Medium, *n* = 13 in AGS, *n* = 12 in SW480. *P < 0.05, **P < 0.01, ***P < 0.001. ^#^Indicates significant differences in V_max_ (Ctr vs. AGS in 1 Hz; Ctr vs. AGS, Ctr vs. SW480, Medium vs. AGS, Medium vs. SW480 in 2 Hz; Ctr vs. AGS in 3 Hz), APA (Ctr vs. AGS, Ctr vs. SW480, Medium vs. AGS, Medium vs. SW480 in 1, 2, and 3 Hz), APD10 (Ctr vs. AGS, Medium vs. AGS in 1, 2, and 3 Hz).

To examine possible effects of cancer cells on the electrophysiological characteristics of cardiomyocytes, the AP of hiPSC-CMs were measured after cancer cell secretion treatment (Figure 1). The maximum depolarization velocity (V_max_) and the action potential amplitude (APA) were reduced and the action potential duration at 10% repolarization (APD10) prolonged in AGS group compared with control (without cancer cell medium) and medium (fresh medium without cancer cells) groups. APA was also decreased in SW480 group compared with control group. There was a decreasing trend in V_max_ and an increasing trend in APD10 in SW480 group. In addition, the effects of cancer cell secretion on AP paced at different frequencies were examined. No frequency-dependence was detected.

To examine possible difference among different cell lines, the experiments were repeated under the same conditions in hiPSC-CMs from other two cell lines. Indeed, effects of cancer secretion in both cell lines were similar to those detected in aforementioned experiments (Supplementary Figure 5), indicating that cancer cell secretion caused similar changes in cell lines from different donors.

**Figure 2:**
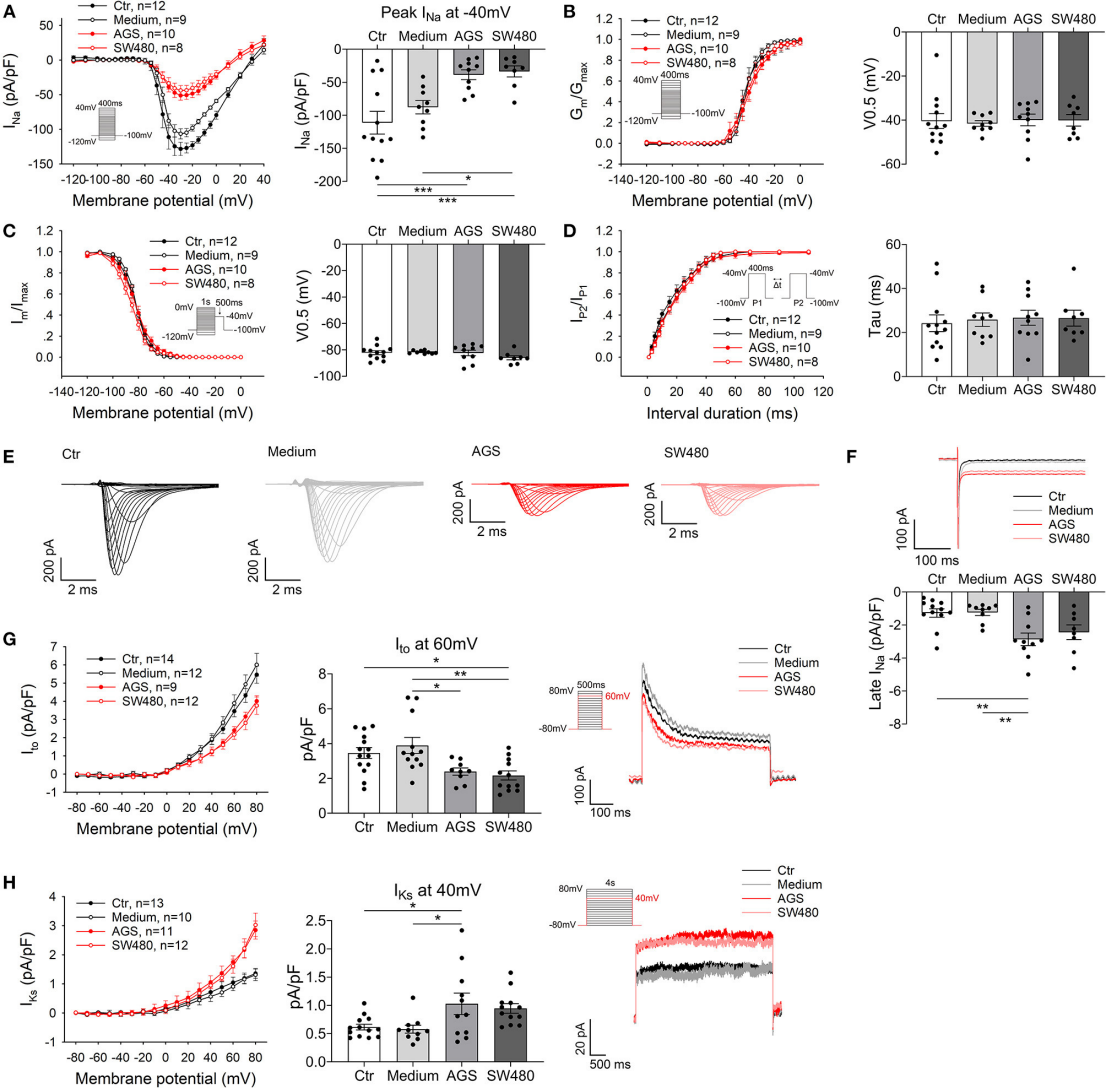
Alterations of ion channel currents caused by cancer cell secretion. The Na^+^ currents (peak I_Na_ and late I_Na_), transient outward current (I_to_) and slowly activating delayed rectifier K^+^ current (I_Ks_) were recorded in hiPSC-CMs cultured with AGS and SW480 cancer cell media. **(A)** I–V curves of peak I_Na_. **(B)** Activation curves of peak I_Na_. **(C)** Inactivation curves of peak I_Na_. **(D)** Time course curves of recovery from inactivation of peak I_Na_. **(E)** Representative traces of I_Na_ in Ctr, medium, AGS, and SW480 groups. **(F)** Representative traces and mean values of late I_Na_ at −40 mV, which was measured at 200 ms of the testing pulse. **(G)** I–V curve (left panel) of I_to_, I_to_ at 60 mV (middle panel) and traces of I_to_ (right panel). **(H)** I–V curve (left panel) of I_Ks_, I_Ks_ at 40 mV (middle panel) and traces of I_Ks_ (right panel). “Ctr” represents data from hiPSC-CMs without medium of cancer cells. “Medium” represents data from hiPSC-CMs with addition of fresh medium for cancer cells. “AGS” represents data from hiPSC-CMs with addition of cultured medium of AGS cancer cells. “SW480” represents data from hiPSC-CMs with addition of cultured medium of SW480 cancer cells. Data are presented as mean ± SEM and analyzed by one-way ANOVA. Cell numbers: peak and late I_Na_: *n* = 12 in Ctr, *n* = 9 in Medium, *n* = 10 in AGS, *n* = 8 in SW480; I_to_: *n* = 14 in Ctr, *n* = 12 in Medium, *n* = 9 in AGS, *n* = 12 in SW480; I_Ks_: *n* = 13 in Ctr, *n* = 10 in Medium, *n* = 11 in AGS, *n* = 12 in SW480. *P < 0.05, **P < 0.01, ***P < 0.001.

Next, different ion channel currents were measured. The peak I_Na_ was significantly reduced in AGS and SW480 (Figures 2A,E) but the activation, inactivation and recovery curves of peak I_Na_ were not significantly changed (Figures 2B–D). The TTX-sensitive late I_Na_ was increased (Figure 2F). The Na/Ca exchanger current (I_NCX_, Supplementary Figure 6) and the L-type calcium channel current (I_Ca−L_, Supplementary Figure 7) were similar in all the experimental groups. The transient outward current I_to_, the 4-AP (an I_to_ blocker) sensitive current, declined in AGS and SW480 groups (Figure 2G). I_Kr_ (HERG channel current) slightly increased (Supplementary Figure 8), while I_Ks_ (slowly activating delayed rectifier K^+^ current) was significantly higher in the cancer groups (Figure 2H). I_K1_ (inward rectifier K^+^ current) displayed no difference in all groups (Supplementary Figure 9).

**Figure 3:**
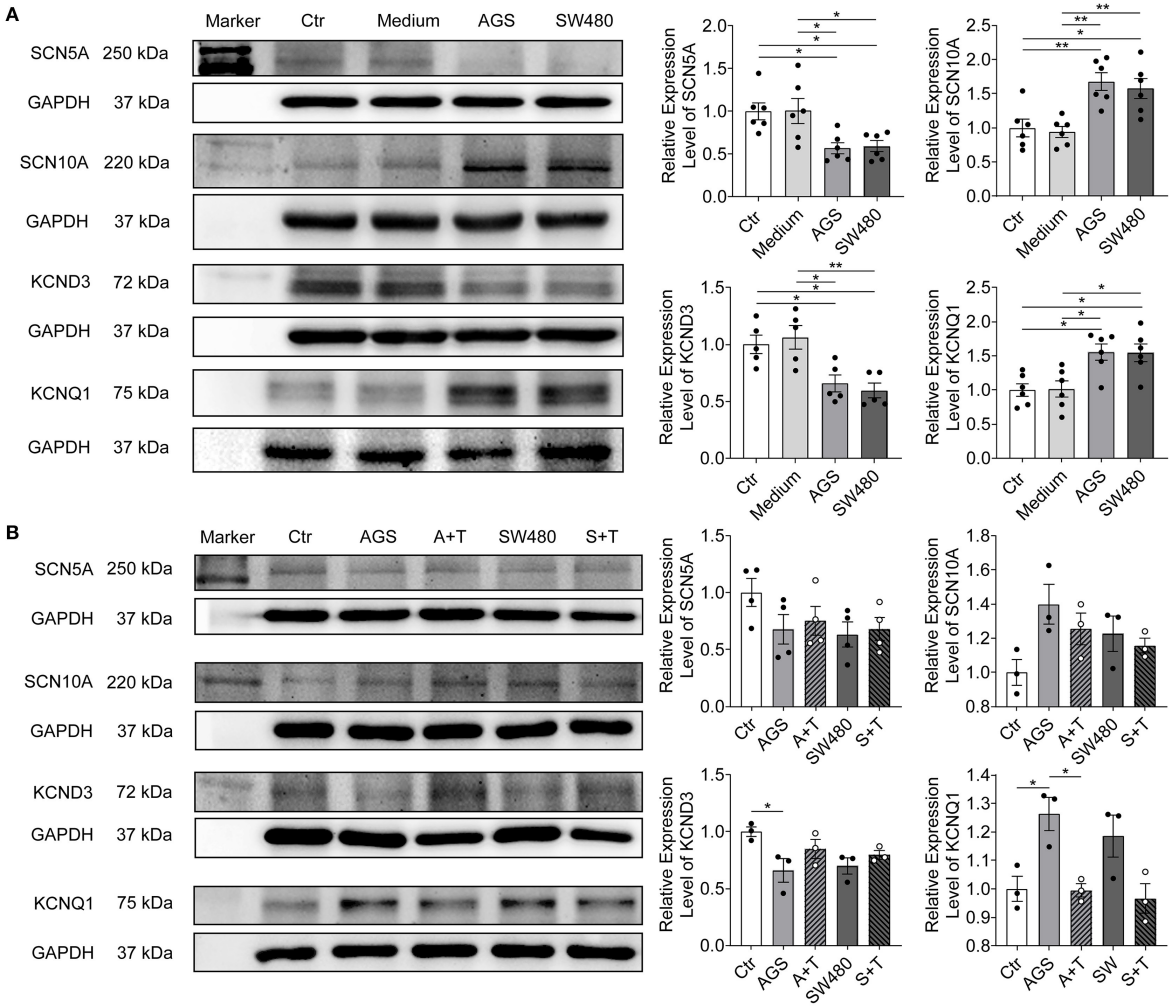
Protein expression levels of SCN5A, SCN10A, KCND3, and KCNQ1 in hiPSC-CMs cultured with AGS and SW480 cancer cell media and after overexpression of TET1. **(A)** Protein bands and statistical analysis of relative expression level of SCN5A, SCN10A, KCND3, and KCNQ1. **(B)** Protein bands and statistical analysis of relative expression level of SCN5A, SCN10A, KCND3, and KCNQ1 after overexpression of TET1. “Ctr” represents data from hiPSC-CMs without medium of cancer cells. “Medium” represents data from hiPSC-CMs with addition of fresh medium for cancer cells. “AGS” represents data from hiPSC-CMs with addition of cultured medium of AGS cancer cells. “SW480” represents data from hiPSC-CMs with addition of cultured medium of SW480 cancer cells. “A+T” represents data from hiPSC-CMs with addition of cultured medium of AGS cancer cells and transfected with 1 μg TET1 plasmid DNA. “S+T” represents data from hiPSC-CMs with addition of cultured medium of SW480 cancer cells and transfected with 1 μg TET1 plasmid DNA. Data are presented as mean ± SEM and analyzed by one-way ANOVA. Experiment numbers: **(A)** SCN5A: *n* = 6, SCN10A: *n* = 6, KCND3: *n* = 5, KCNQ1: *n* = 6; **(B)** SCN5A: *n* = 4, SCN10A: *n* = 3, KCND3: *n* = 3, KCNQ1: *n* = 3. *P < 0.05, **P < 0.01.

Then, we checked the protein expression level of the ion channels. The SCN5A and KCND3 protein levels were decreased, whereas SCN10A and KCNQ1 levels were elevated (Figure 3A). CACNA1C, Na^+^/Ca^2+^ exchanger NCX, HERG, and KCNJ2 levels were not changed (Supplementary Figure 10). All the results were in accordance with PCR assays (Supplementary Figure 4) and current measurements (Figure 2, Supplementary Figures 6–9).

### Cancer Cell Secretion Enhanced the DNA Methylation of Whole Genome and in Promoters of Ion Channel Genes

**Figure 4:**
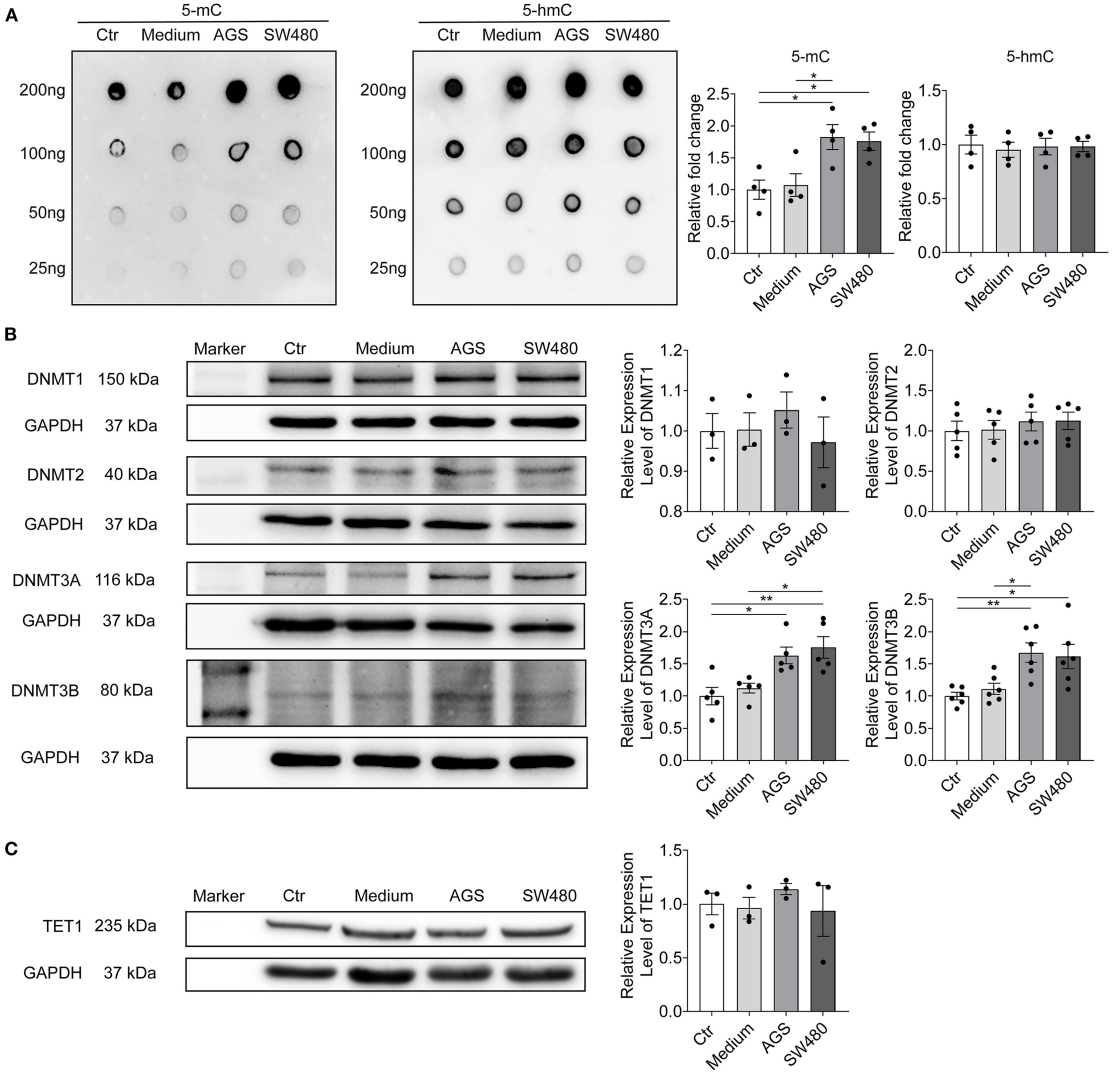
Cancer cell secretion elevated the whole genome DNA methylation and DNMTs expression. The whole genome DNA methylation and protein expression levels of DNMTs and TET1 were analyzed in hiPSC-CMs cultured with AGS and SW480 cancer cell media. **(A)** Whole genome DNA methylation level detected by 5-mC antibody and DNA demethylation level detected by 5 hmC antibody and their statistical analysis. **(B)** Protein bands and statistical analysis of relative expression level of DNMT1, 2, 3A, and 3B. **(C)** Protein bands and statistical analysis of relative expression level of TET1. “Ctr” represents data from hiPSC-CMs without medium of cancer cells. “Medium” represents data from hiPSC-CMs with addition of fresh medium for cancer cells. “AGS” represents data from hiPSC-CMs with addition of cultured medium of AGS cancer cells. “SW480” represents data from hiPSC-CMs with addition of cultured medium of SW480 cancer cells. Data are presented as mean ± SEM and analyzed by one-way ANOVA. Experiment numbers: **(A)** *n* = 4; **(B)** DNMT1: *n* = 3, DNMT2: *n* = 5, DNMT3A: *n* = 5, DNMT3B: *n* = 6, **(C)** TET1: *n* = 3. *P < 0.05, **P < 0.01.

To examine the potential epigenetic mechanism by which GI cancers cause ion channel dysfunction, the levels of whole genome DNA methylation and demethylation were both measured. The dot blotting results showed that the level of 5-methylcytosine (5-mC, indicating DNA methylation) but not 5-hydroxymethylcytosine (5-hmC, indicating DNA demethylation) was higher in AGS and SW480 groups (Figure 4A).

**Figure 5:**
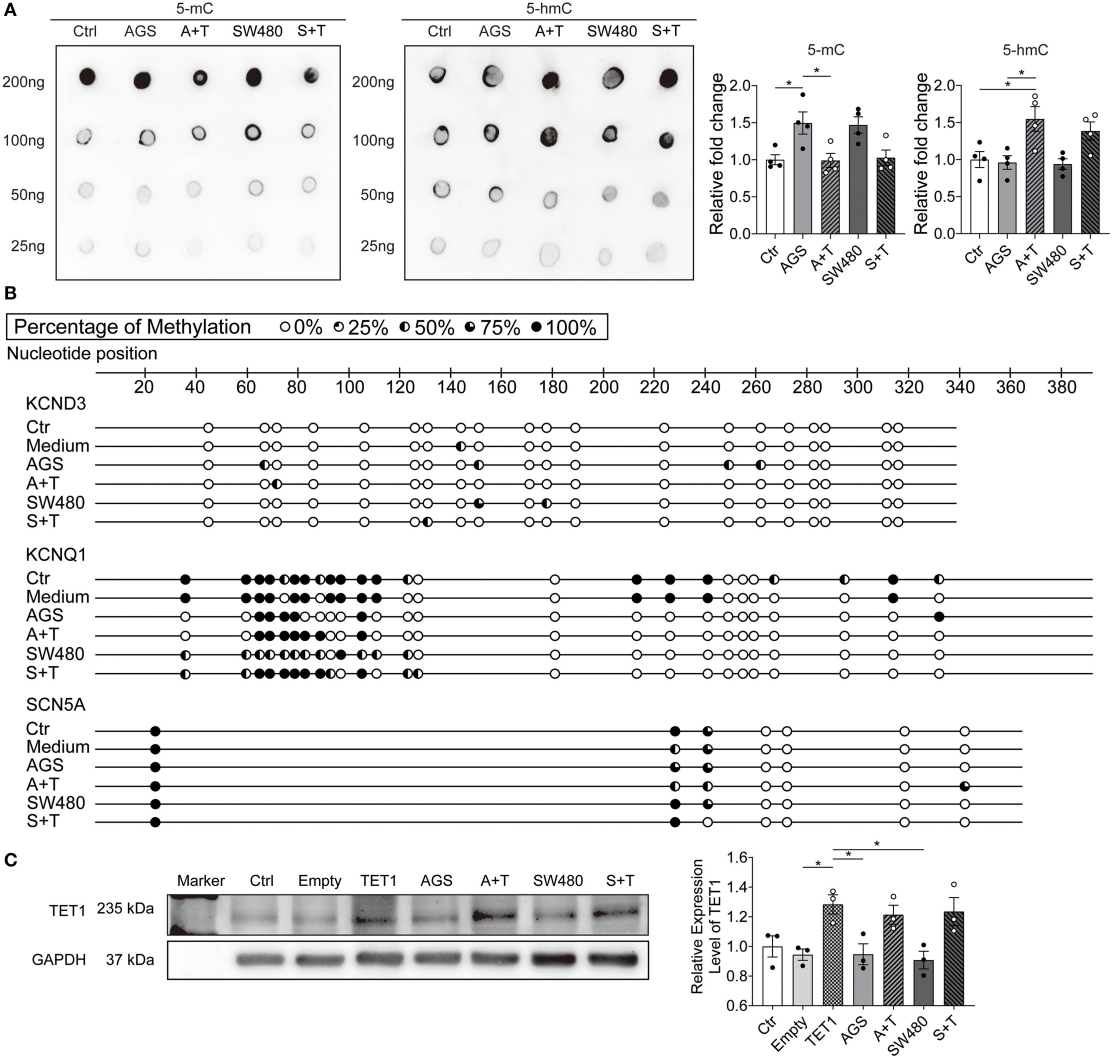
Overexpression of TET1 prevented effects of cancer cell secretion on DNA methylation. The hiPSC-CMs were transfected with 1 μg TET1 plasmid DNA or the empty plasmid. The successful overexpression of TET1 was confirmed by the protein level of TET1 analyzed by western blotting. The whole genome DNA methylation level, DNA methylation level in CpG island in promoter regions of KCND3, KCNQ1, and SCN5A genes and protein expression levels of TET1 were assessed in hiPSC-CMs cultured with AGS and SW480 cancer cell media after overexpression of TET1. **(A)** Whole genome DNA methylation level detected by 5-mC antibody and DNA demethylation level detected by 5-hmC antibody and their statistical analysis. **(B)** DNA methylation level in CpG island in promoter regions of KCND3, KCNQ1, and SCN5A genes. **(C)** Protein bands and statistical analysis of relative expression level of TET1. “Ctr” represents data from hiPSC-CMs without transfection. “Empty” represents data from hiPSC-CMs transfected with empty plasmid. “TET1” represents data from hiPSC-CMs transfected with 1 μg TET1 plasmid DNA. “Medium” represents data from hiPSC-CMs with addition of fresh medium for cancer cells. “AGS” represents data from hiPSC-CMs with addition of cultured medium of AGS cancer cells without transfection. “A+T” represents data from hiPSC-CMs with addition of cultured medium of AGS cancer cells and transfected with 1 μg TET1 plasmid DNA. “SW480” represents data from hiPSC-CMs with addition of cultured medium of SW480 cancer cells without transfection. “S+T” represents data from hiPSC-CMs with addition of cultured medium of SW480 cancer cells and transfected with 1 μg TET1 plasmid DNA. Data are presented as mean ± SEM and analyzed by one-way ANOVA. Experiment numbers: **(A)** *n* = 4; **(C)** TET1: *n* = 3. *P < 0.05.

Next, we evaluated the methylation levels of CpG islands in promoters of KCND3, KCNQ1, and SCN5A channel genes (Figure 5B). The methylation levels of CpG islands of KCND3 were higher in cancer secretion groups, while the methylation levels of KCNQ1 were lower than those in control and medium groups. No difference of methylation level of CpG islands in SCN5A gene was detected among all groups.

DNA Methyltransferases (DNMTs) are the main enzymes responsible for DNA methylation. We further checked the protein levels of DNMT1, DNMT2, DNMT3A, and DNMT3B (Figure 4B). DNMT3A and 3B levels were increased significantly in cancer cell secretion groups, in agreement with the elevated methylation in DNA. The level of TET1 enzyme, which is responsible for DNA demethylation, was not changed by cancer cell secretion (Figure 4C).

To prove that the observed changes of ion channel expression induced by cancer cell secretion were related to the enhanced DNA methylation, we attempted to reduce the methylation by overexpression of TET1. After transfection, the TET1 plasmid but not the empty plasmid increased the protein level of TET1 in hiPSC-CMs, independent of cancer secretions (Figure 5C). Under this condition, the level of 5-mC was decreased and 5-hmC was increased in hiPSC-CMs treated with cancer secretions (Figure 5A). The changes of methylation levels of CpG islands of promoters of KCND3 channel genes were also rescued by TET1 expression (Figure 5B).

To assess whether the changes in DNA methylation level induced by cancer cell secretion were responsible for ion channel dysfunction, the protein expression levels of KCND3, KCNQ1, SCN5A, and SCN10A were evaluated in cells overexpressed with TET1 reducing the DNA methylation level. Indeed, the KCND3 and KCNQ1 changes were rescued after overexpression of TET1. Surprisingly, the expression of Na^+^ channels (SCN5A and SCN10A) was not changed (Figure 3B).

**Figure 6:**
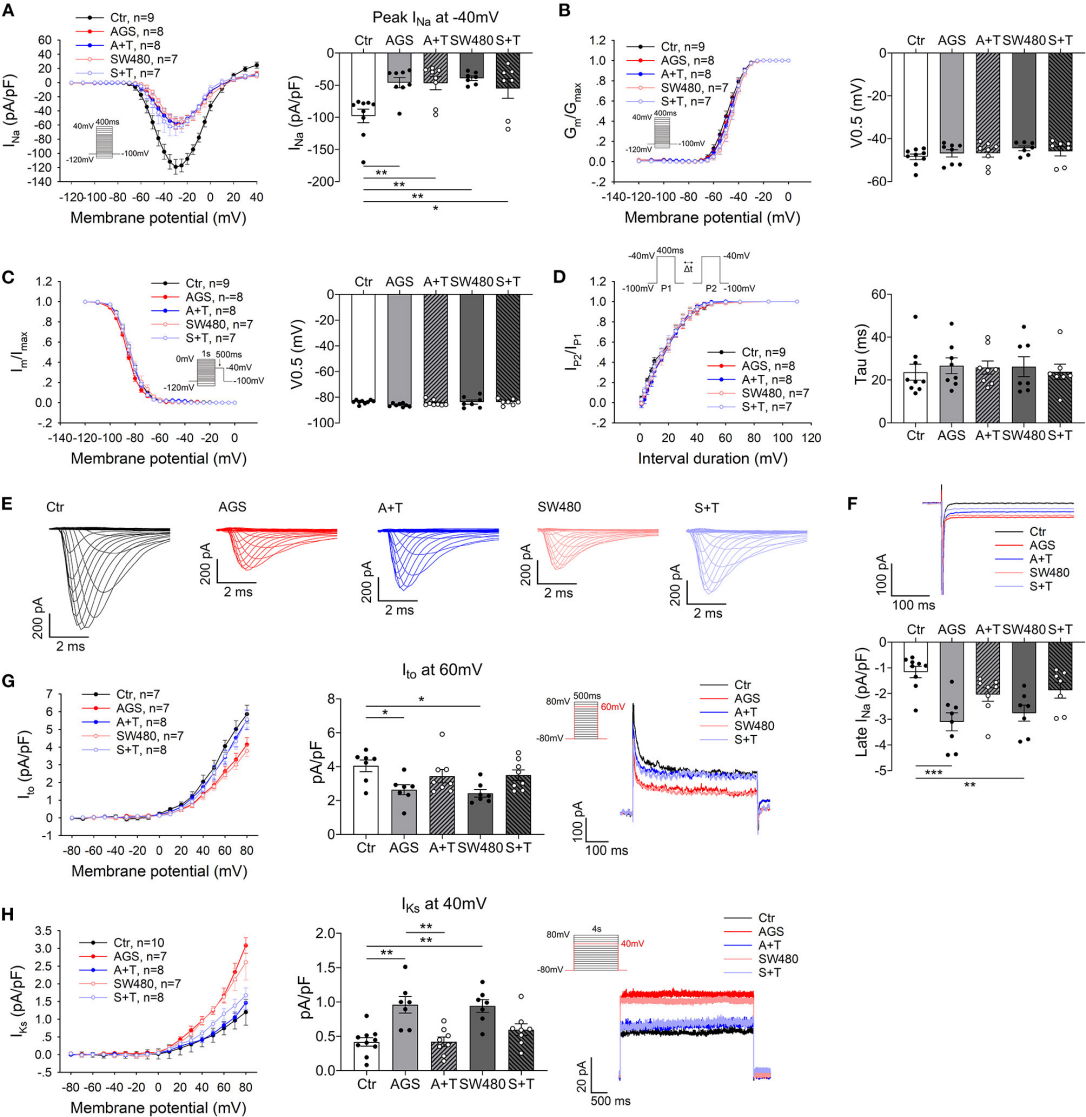
Overexpression of TET1 prevented effects of cancer cell secretion on ion channel currents. The hiPSC-CMs were transfected with 1 μg TET1 plasmid DNA or the empty plasmid. The Na^+^ currents (peak I_Na_ and late I_Na_), transient outward current (I_to_) and slowly activating delayed rectifier K^+^ current (I_Ks_) were assessed in hiPSC-CMs cultured with AGS and SW480 cancer cell media after overexpression of TET1. **(A)** I–V curves of peak I_Na_. **(B)** Activation curves of peak I_Na_. **(C)** Inactivation curves of peak I_Na_. **(D)** Time course curves of recovery from inactivation of peak I_Na_. **(E)** Representative traces of I_Na_ in Ctrl, medium, AGS, and SW480 groups. **(F)** Representative traces and mean values of late I_Na_ at −40 mV. **(G)** I–V curve, I_to_ at 60 mV and traces of I_to_. **(H)** I–V curve, I_ks_ at 40 mV and traces of I_ks_. “Ctr” represents data from hiPSC-CMs with addition of un-cultured medium for cancer cells. “AGS” represents data from hiPSC-CMs with addition of cultured medium of AGS cancer cells. “A + T” represents data from hiPSC-CMs with addition of cultured medium of AGS cancer cells and transfected with 1 μg TET1 plasmid DNA. “SW480” represents data from hiPSC-CMs with addition of cultured medium of SW480 cancer cells. “S + T” represents data from hiPSC-CMs with addition of cultured medium of SW480 cancer cells and transfected with 1 μg TET1 plasmid DNA. “Data are presented as mean ± SEM and analyzed by one-way ANOVA. Cell numbers: peak and late I_Na_: *n* = 9 in Ctr, *n* = 8 in AGS, *n* = 8 in A + T, *n* = 7 in SW480, *n* = 7 in S + T; I_to_: *n* = 7 in Ctr, *n* = 7 in AGS, *n* = 8 in A + T, *n* = 7 in SW480, *n* = 8 in S + T; I_Ks_: *n* = 10 in Ctr, *n* = 7 in AGS, *n* = 8 in A + T, *n* = 7 in SW480, *n* = 8 in S + T. *P < 0.05, **P < 0.01, ***P < 0.001.

Finally, we examined the influences of DNA methylation level on ion channel functions. When TET1 was overexpressed (DNA methylation level was reduced) in hiPSC-CMs treated with cancer cell secretion, the peak I_Na_ and late I_Na_ were not changed (Figures 6A–F). However, both I_to_ and I_Ks_ were rescued to an extent similar to the control group (Figures 6G,H).

### Cancer Cell Secretion Regulates DNA Methylation of Cardiomyocytes Through PI3K/Akt Pathway

To determine whether the effects on cardiomyocytes was cancer cell dependent, we applied endothelial cell medium, HEK293 cell medium, and their medium without cells to hiPSC-CMs. The dot blotting revealed no significant effects (Supplementary Figure 11).

**Figure 7:**
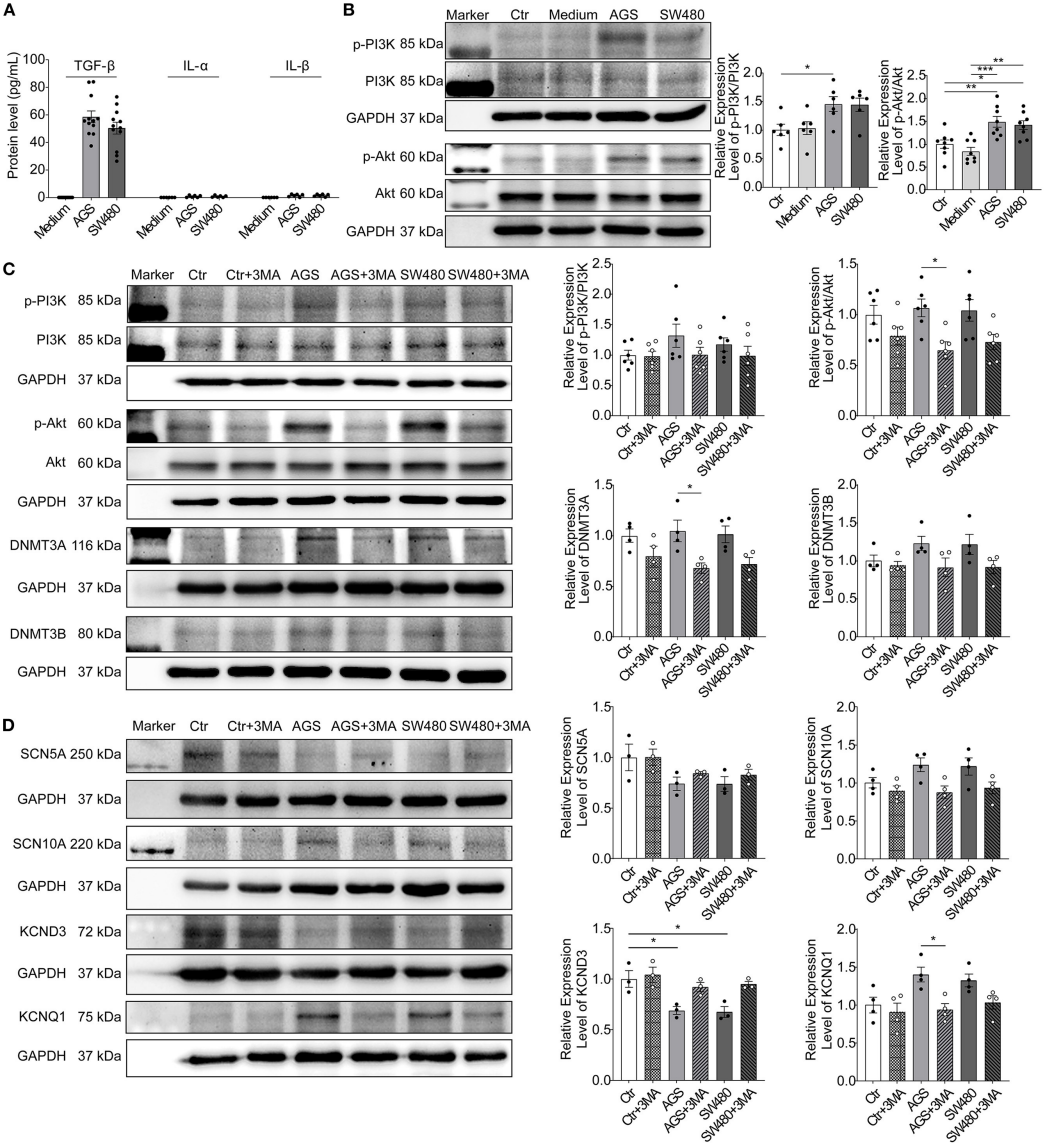
TGF-β/PI3K/Akt signaling contributed to effects of cancer cell secretions. Protein levels of IL-1α, IL-1β, and TGF-β in cancer cell media and activation of PI3K/Akt signaling were analyzed in hiPSC-CMs cultured with AGS and SW480 cancer cell media. **(A)** Protein levels of IL-1α, IL-1β, and TGF-β in cancer cell media detected by ELISA. **(B)** Protein bands and statistical analysis of relative expression level of p-PI3K/PI3K and p-Akt/Akt. **(C)** Protein bands and statistical analysis of relative expression level of p-PI3K/PI3K, p-Akt/Akt, DNMT3A, and DNMT3B after treatment of 3MA. **(D)** Protein bands and statistical analysis of relative expression level of SCN5A, SCN10A, KCND3, and KCNQ1 after treatment of 3MA. “Ctr” represents data from hiPSC-CMs without medium of cancer cells. “Ctr + 3MA” represents data from hiPSC-CMs without medium of cancer cells but treated with 3MA. “Medium” represents data from hiPSC-CMs with addition of fresh medium for cancer cells. “AGS” represents data from hiPSC-CMs with addition of cultured medium of AGS cancer cells. “AGS + 3MA” represents data from hiPSC-CMs with addition of cultured medium of AGS cancer cells and treated with 3MA. “SW480” represents data from hiPSC-CMs with addition of cultured medium of SW480 cancer cells. “SW480 + 3MA” represents data from hiPSC-CMs with addition of cultured medium of SW480 cancer cells and treated with 3MA. Data are presented as mean ± SEM and analyzed by one-way ANOVA. Experiment numbers: **(A)** TGF-β: *n* = 12, IL-1α: *n* = 6, IL-1β: *n* = 6; **(B)** p-PI3K/PI3K: *n* = 6, p-Akt/Akt: *n* = 8; **(C)** p-PI3K/PI3K: *n* = 6, p-Akt/Akt: *n* = 6, DNMT3A: *n* = 4, DNMT3B: *n* = 4. **(D)** SCN5A: *n* = 3, SCN10A: *n* = 4, KCND3: *n* = 3, KCNQ1: *n* = 4. *P < 0.05, **P < 0.01, ***P < 0.001.

To further explore ingredients in the cancer culture medium and their mechanisms for the observed effects on cardiomyocytes, we examined the interleukin-1α (IL-1α), IL-1β, transforming growth factor-β (TGF-β) levels in medium by ELISA. The amount of TGF-β was significantly higher than IL-1α and IL-1β in AGS and SW480 cancer cell media (Figure 7A). Then, we checked whether the increase of DNMT3a and DNMT3b was mediated by TGF-β through phosphoinositide 3-kinase/protein kinase B (PI3K/Akt) signaling. The levels of phosphorylated PI3K and Akt were elevated in cancer cell secretion groups (Figure 7B). Furthermore, after using 3-Methyladenine (3-MA), an inhibitor of PI3K, the increased levels of phosphorylated PI3K and Akt were decreased, and DNMT3A, DNMT3B, KCND3, and KCNQ1 levels were also rescued compared with AGS and SW480 cancer cell media treated cardiomyocytes (Figures 7C,D). To confirm the effect of TGF-β on the ion channel expression, we treated the cardiomyocytes with TGF-β. The protein levels of SCN5A and KCND3 decreased and SCN10A and KCNQ1 increased, similar to the levels in AGS and SW480 cancer cell media treated cells (Supplementary Figure 12).

## Discussion

In the present study, we investigated for the first time the effects of cancer cell secretion without chemotherapy on ion channel function and the underlying mechanism using hiPSC-CMs.

Generally, cardiac side effects of chemotherapy include cardiac systolic dysfunction, cardiac ischemia, arrhythmias, pericarditis, which are associated with cytotoxic effects of chemotherapy (16). Arrhythmias have been described in many patients with chemotherapy, which might be the direct result of cardiotoxicity, or caused by cardiac ischemia or related to metabolic changes associated with the use of chemotherapy. Repolarization abnormalities with QT prolongation, which may be a substrate for arrhythmias, were frequently detected in patients with chemotherapy (17). Repolarization abnormalities might be the direct result of ion channel block or the result of changes in hepatic metabolism caused by chemotherapy and a change in clearance of other drugs associated with QT prolongation (16, 18, 19). We found that cancer cell secretion can prolong APD10. In addition, we observed that cancer cell secretion can suppress the V_max_ and APA of APs. Although the mild prolongation of APD10 and reduction of V_max_ may not be enough to directly cause arrhythmias, their possible contribution to arrhythmogenesis cannot be excluded. It is well-known that the prerequisite condition for the occurrence of arrhythmias is multifactorial, e.g., a substrate and a trigger is usually required. In cancer patients, the cardiomyocytes can be affected by cancer secretion, by cancer cell-induced dysfunction of other systems like nerve/endocrine system, or by drugs, each of which may affect some ion channel function. The effects of different factors may be added to increase the chance for occurrence of arrhythmias. Therefore, the effects of cancer cell secretion on I_to_ and I_Na_ as well as APs (reduction of V_max_ and prolongation of APD10) may contribute to occurrence of arrhythmias in cancer patients.

To explore the reason for AP changes, we analyzed the ion channel currents that contributed to the AP and found that the peak I_Na_ was decreased significantly by cancer cell secretion. However, the gating kinetics Na channels including the activation, inactivation, and recovery from inactivation were not changed, suggesting that the reduction of peak I_Na_ resulted from reduced the channel proteins rather than gating changes. This is consistent with the PCR and western blot data showing reductions of both mRNA and protein level. The reduction of peak I_Na_ can be explained by the downregulation of SCN5A, whereas the enhancement of late I_Na_ may result from the upregulation of SCN10A, which has been shown to contribute to late I_Na_ (20, 21). The peak I_Na_ predominantly contributes to the depolarization of AP. The decrease of V_max_ and APA in cardiomyocytes by cancer cell secretion can be explained by the decreased peak I_Na_. The enhanced late I_Na_ may prolong APD. The I_to_ was decreased but the I_Ks_ was increased by cancer cell secretion. The changes of both I_to_ and I_Ks_ also resulted from changes of their gene and protein expression. We also measured I_Ca−L_, I_NCX_, I_Kr_, and I_K1_ but failed to detect any significant differences among these groups. The decrease in I_to_ may be the cause for the prolongation of APD10 since this phase is mainly determined by I_to_. The increase in I_Ks_ should shorten APD, especially APD90. However, in the current study, APD50 and APD90 were not significantly changed by cancer cell secretion. This probably resulted from the counteraction of increased late I_Na_ and I_Ks_, both of which can influence APD, but in opposite directions. I_Ca−L_ and I_K1_ as well as I_Kr_ are also important for determining APD. Given that these three currents were not significantly changed by cancer cell secretion, their contributions to the changes of APs are negligible.

Taken together, the cancer cell secretions can change some cardiac ion channel currents and the current changes were caused by changes of gene expression. The next question is how the gene expression was regulated by cancer cells.

DNA methylation is an important epigenetic regulation of genes. Enhanced methylation may suppress gene expression. Although the reasons for the downregulation of SCN5A and upregulation of SCN10A are unknown (no change in methylation level in SCN5A gene was detected and no CpG island in promoter of SCN10A gene were found), the enhanced methylation in KCND3 gene may be a reason for the downregulation of I_to_ channels and the attenuated methylation in KCNQ1 gene is probably a reason for the upregulation of I_Ks_ channels. The enhanced methylation level in whole genome DNA and I_to_ gene can be explained by upregulation of DNMT3A and DNMT3B. However, the underlying mechanism by which the level of DNMTs was regulated was still unclear. It is believed that cancer cells could interact with the host cells and regulate their gene expression (22). Cancer cells might contribute to the DNMTs dysregulation in cardiomyocytes by secretion of cytokines or extracellular vesicles. GI cancer cells can secrete cytokines such as IL-1β, IL-6, TGF-β, etc. (23, 24). These cytokines such as IL-1β and TGF-β1 can activate cellular signaling pathways and lead to changes in expression levels of DNMTs (25, 26). It was reported that IL-1β could induce an increase in the DNMT activity via NF-κB pathway in gastric cancer cells (27) and TGF-β1 could up-regulate the expression of DNMT1 and 3a though different mechanisms including activation of the PI3K/Akt pathway (28). On the other hand, TGF-β signaling might also have direct regulatory effect on ion channel expression. TGF-β signaling was reported to regulates Ca^2+^ ion channel expression and activities (29). In the present study, we detected the levels of IL-1α, IL-1β, and TGF-β in AGS and SW480 cancer cell media and found that TGF-β level was increased significantly in both cancer cell media. We further found that the PI3K/Akt signaling pathway was activated as evidenced by increased levels of the actively phosphorylated form of PI3K and Akt protein in AGS and SW480 group cardiomyocytes. Moreover, 3-MA, a PI3K inhibitor could reduce the activation of PI3K/Akt signaling pathway and reverse the DNMT3a and 3b levels in AGS and SW480 cancer cell media treated cardiomyocytes. Thus, TGF-β and activation of PI3K/Akt pathway might be one of the underlying mechanisms of cancer-induced ion channel gene regulation and cardiophysiological changes.

## Study Limitations

Our study has certain limitations. Firstly, the cultured cancer cell media have different factors which might regulate DNA methylation or directly affect the expression and activities of ion channels of cardiomyocytes. Factors besides TGF-β, IL-1a, and IL-1β have not been investiagted in this study. Secondly, the mechanisms underlying the downregulation of SCN5A and upregulation of SCN10A as well as the reduced methylation of I_Ks_ gene by cancer cell secretions were not revealed. Thirdly, only two cancer cell lines were used for the study, whether other types of cancer cells exert similar or different effects on cardiomyocytes is till an open question. In addition, the immaturity (embryonic feature) of hiPSC-CMs is a well-known limitation of hiPSC-CMs. The possibility that hiPSC-CMs show experimental results different from that obtained in native human cardiomyocytes cannot be excluded.

## Conclusions

Our study demonstrates that cancer cell secretions can influence cardiac ion channel expresion and function. The underlying mechanism was at least partially the increased DNA methylation in ion channel genes by upregulation of DNMTs through TGF-β/PI3K/Akt signaling. The results indicated that ion channel dysfunction caused by cancer cell secretion may contribute to arrhythmogenesis in cancer patients. Investigating the regulatory signaling, especially the epigenetic modifications of ion channels, may provide opportunities for discovering new therapeutic targets for treating arrhythmias in cancer patients.

## Data Availability Statement

The original contributions presented in the study are included in the article/Supplementary Material, further inquiries can be directed to the corresponding author.

## Ethics Statement

The studies involving human participants were reviewed and approved by the Ethics Committee of Medical Faculty Mannheim, Heidelberg University and the Ethics Committee of the University Medical Center Göttingen. The patients/participants provided their written informed consent to participate in this study.

## Author Contributions

XZ, RZ, and FZ designed the project and drafted the manuscript. RZ and FZ carried out most part of the experiments. LC and EB provided the cell lines. RZ, FZ, ZY, YL, QX, HL, SL, and IE-B contributed to the cell culture and experimental materials, statistical analyses, and results interpretation. IA and MB shared the research administration and revised the manuscript. All authors edited and approved the final version of the manuscript.

## Funding

This study was supported by the DZHK (German Center for Cardiovascular) and the BMBF (German Ministry of Education and Research).

## Conflict of Interest

The authors declare that the research was conducted in the absence of any commercial or financial relationships that could be construed as a potential conflict of interest.

## Publisher's Note

All claims expressed in this article are solely those of the authors and do not necessarily represent those of their affiliated organizations, or those of the publisher, the editors and the reviewers. Any product that may be evaluated in this article, or claim that may be made by its manufacturer, is not guaranteed or endorsed by the publisher.
