# Supplementary material for: Regulation of Ion Channel Function in Human-Induced Pluripotent Stem Cell-Derived Cardiomyocytes by Cancer Cell Secretion Through DNA Methylation
Source: Front Cardiovasc Med. 2022 Feb 21;9:839104. doi: 10.3389/fcvm.2022.839104 (PMC8899119; doi:10.3389/fcvm.2022.839104)
Supplement: Supplementary file 1 [file Data_Sheet_1.pdf]

SUPPLEMENTARY MATERIAL

Regulation of ion channel function in human-induced pluripotent stem cell-derived cardiomyocytes by cancer cell secretion through DNA methylation

Rujia Zhong[1]*, Feng Zhang[1]*, Zhen Yang[1], Yingrui Li[1], Qiang Xu[1], Huan Lan[3], Siegfried Lang[1,2], Lukas Cyganek[4,5], Elke Burgermeister[6], Ibrahim El-Battrawy[1,2], Xiaobo Zhou[1,2,3], Ibrahim Akin[1,2] and Martin Borggrefe[1,2]

[1]First Department of Medicine, Medical Faculty Mannheim, University Medical Centre Mannheim (UMM), University of Heidelberg, Mannheim, Germany
[2]DZHK (German Center for Cardiovascular Research), Partner Site, Heidelberg-Mannheim, Mannheim, Germany
[3]Key Laboratory of Medical Electrophysiology of Ministry of Education and Medical Electrophysiological Key Laboratory of Sichuan Province, Institute of Cardiovascular Research, Southwest Medical University, Luzhou, Sichuan, China
[4]Stem Cell Unit, Clinic for Cardiology and Pneumology, University Medical Center Göttingen, Göttingen, Germany
[5]DZHK (German Center for Cardiovascular Research), Partner Site, Göttingen, Göttingen, Germany
[6]Second Department of Medicine, Faculty of Medicine, University Medical Centre Mannheim (UMM), University of Heidelberg, Mannheim, Germany

*These authors contributed equally to this work.
Drs Akin and Borggrefe share the senior authorship.

Address for correspondence:
Xiaobo Zhou, MD
First Department of Medicine, Faculty of Medicine, University Medical Centre Mannheim, University of Heidelberg, Theodor-Kutzer-Ufer 1-3, 68167 Mannheim, Germany. **Tel**: +49-621-3831448, **Fax**: +49-621-3831989, **E-mail**: Xiaobo.zhou@medma.uni-heidelberg.de

Funding
This study was supported by the DZHK (German Center for Cardiovascular) and the BMBF (German Ministry of Education and Research).
Conflicts of interests: None
Key words - Cancer cell secretion; human-induced pluripotent stem cell-derived cardiomyocyte; ion channel; DNA methylation; arrhythmia.

Supplementary methods

Differentiation and culture of hiPSCs

The directed differentiation of hiPSCs into cardiomyocytes (hiPSC-CMs) was initiated at 80–90% confluence in 24-well plates coated with Matrigel. The cardio maintenance medium composed of RPMI 1640 with Glutamax and HEPES (Thermo Fisher Scientific), 2% B27 (Thermo Fisher Scientific), 0.2mg/ml L-ascorbic acid 2-phosphate (Sigma-Aldrich), 1% Sodium Pyruvate (Gibco), and 1% Pen/Strep (Thermo Fisher Scientific). For cardio differentiation, the hiPSCs were sequentially treated with cardio maintenance medium adding 1μM CHIR99021 (StemMACS), 5ng/ml BMP4 (R&D Systems), 9ng/ml activin A (R&D Systems), 5ng/ml FGF (Miltenyi Biotec) for 3 days and then 5μM IWP2 (StemCell Technologies) for about 8-10 days with the cardio maintenance medium. Differentiated cells were glucose-starved and supplemented with 5mM Sodium Lactate Solution (Sigma-Aldrich), 50mM 2-mercaptoethanol (Gibco) with RPMI 1640 without glucose nor glutamine to metabolically select hiPSC-CMs around day 13-15. The iPSC-CMs were cultured in maintenance media at least to day 50-60 for further experience. Cardiomyocytes were dissociated from 24-well plates for biological studies or plated on Matrigel-coated 3.5cm petri dishes for patch-clamp measurements. For preparing the single cell from 24-well plate to dishes.

Manual cell counting and MTT assay

Manual cell counting was carried out with a hemocytometer and a microscope, using 0.4% trypan blue-treated cell suspension. The number of cells in all 4 outer squares were counted and then divided by 4 (the mean number of cells per square). The number of cells per milliliter of suspension equaled to the number of the living cells per square multiplied by 10^4.

MTT assay is a quantitative colorimetric method, which allows evaluation of cell viability and proliferation, and cytotoxicity of different compounds. The

method is based on enzyme reduction of MTT tetrazolium salt (3-(4,5-dimethylthiazolyl)-2,5-diphenyltetrazolium bromide) to its dark blue insoluble formazan. The hiPSC-CMs around day 50-60 were seeded in 96-well plates (5 × 10^3 cells per well) and cultured for 12h in a cell incubator. Then different concentration of cancer cell media was added for another 48h. The hiPSC-CMs added with fresh cardio culture media were used as a negative control. Then 20μL MTT (5 mg/mL in PBS, Sigma-Aldrich) were added to each well. After 4h incubation, the supernatant was replaced with 150μL of DMSO solution (Sigma-Aldrich). Absorbance OD was measured at 490nm and cell viability was calculated.

Polymerase chain reaction assays

hiPSC-CMs were collected and extracted for total RNA with Total RNA extraction reagent (Qiagen, Hilden, Germany). The cDNA synthesized from total RNA was amplified by qPCR on the Real-time PCR System (Applied Biosystems StepOnePlus™ Real-Time PCR System) using a PCR mix with hot start Taq DNA polymerase and SYBR Green (119405, BIORON, Germany) in the presence of sense and antisense primers (shown in Supplementary table 2). The relative expression levels of each mRNA were calculated as the gene of interest relative to GAPDH which was calculated by the ΔΔCT method, based on the threshold cycle (CT).

Solutions for patch clamp measurements

For AP measurements, the isotonic solution contained 130mmol/L NaCl, 5.9mmol/L KCl, 1.2mmol/L $MgCl_2$, 2.4mmol/L $CaCl_2$, 11mmol/L glucose, and 10 mmol/L HEPES (pH 7.4 (NaOH)). The pipette solution contained 20mmol/L KCl, 110mmol/L K-aspartate, 1mmol/L $MgCl_2$, 0.5mmol/L EGTA, 2mmol/L ATP, 0.5mmol/L GTP, and 10mmol/L HEPES (pH 7.2 (KOH)).

For peak sodium current (I_{Na}) measurements, the bath solution contained

20mmol/L NaCl, 110mmol/L CsCl, 1.8mmol/L $CaCl_2$, 1mmol/L $MgCl_2$, 10mmol/L HEPES, 10mmol/L glucose, and 0.001mmol/L nifedipine (pH 7.4 (CsOH)). Microelectrodes were filled with 10mmol/L NaCl, 135mmol/L CsCl, 2mmol/l $CaCl_2$, 3mmol/L MgATP, 2mmol/L TEA-Cl, 5mmol/L EGTA, and 10mmol/L HEPES (pH 7.2 (CsOH)). For late I_{Na}, the same solutions were used.

The bath solution for L-type calcium channel current ($I_{Ca\text{-}L}$) recordings contained 140mmol/L TEA-Cl, 5mmol/L $CaCl_2$, 1mmol/L $MgCl_2$, 10mmol/L HEPES, 0.003mmol/L E-4031, 0.02mmol/L TTX, and 3mmol/L 4-AP (pH 7.4 (CsOH)). The pipette solution contained 10mmol/L NaCl, 135mmol/L CsCl, 2mmol/L $CaCl_2$, 3mmol/L MgATP, 2mmol/L TEA-Cl, 5mmol/L EGTA, and 10mmol/L HEPES (pH 7.2 (CsOH)).

The bath solution for late I_{Na} contained 135mmol/L NaCl, 20mmol/L CsCl, 1.8mmol/L CaCl2, 1mmol/L MgCl2, 10mmol/L Hepes, 10mmol/L glucose, 0.001mmol/L nifedipine (pH 7.4 (CsOH)). Microelectrodes were filled with 10mmol/L NaCl, 135mmol/L CsCl, 2mmol/L CaCl2, 3mmol/L MgATP, 2mmol/L TEA-Cl, 5mmol/L EGTA and 10mmol/L HEPES (pH7.2 (CsOH)).

The bath solution for Na^+-Ca^{2+} exchanger current (I_{NCX}) measurements contained 135mmol/L NaCl, 10mmol/L CsCl, 2mmol/L $CaCl_2$, 1mmol/L $MgCl_2$, 10mmol/L HEPES, 10mmol/L glucose, 0.01mmol/L nifedipine, 0.1mmol/L niflumic acid, 0.05mmol/L lidocaine, and 0.02mmol/L dihydroouabain (pH 7.4 (CsOH)). Microelectrodes were filled with 10mmol/L NaOH, 150mmol/L CsOH, 2mmol/L $CaCl_2$, 1mmol/L $MgCl_2$, 75mmol/L aspartic acid, and 5mmol/L EGTA (pH 7.2 (CsOH)).

For measuring K^+ channel currents, the bath solution contained 130mmol/L NaCl, 5.9mmol/L KCl, 2.4mmol/L $CaCl_2$, 1.2mmol/L $MgCl_2$, 11mmol/L glucose, and 10mmol/L HEPES (pH 7.4 (NaOH)). The pipette solution contained 126mM KCl, 6mM NaCl, 1.2mM MgCl2, 5mM EGTA, 11mM glucose, 10mM HEPES, and 1mM MgATP (pH 7.4 (KOH)). For the transient outward K+ current (Ito) measurements, 10μM nifedipine, 10μM TTX, and 3μM E-4031 were added in

the bath solution to block $I_{Ca\text{-}L}$, I_{Na}, and I_{Kr}, respectively. For the inward rectifier current (I_{K1}) measurement, 10μM nifedipine and 3μM E-4031 were added. For the slowly activating delayed rectifier K^+ (I_{Ks}) measurements, 10μM nifedipine, 3mM 4-AP, and 10μM TTX were added.

To improve the measurement of the rapidly activating delayed rectifier channels (I_{Kr}), the Cs^+ instead of K^+ ions were used as the charge carrier. External solution for Cs^+ current measurements contains 140mmol/L CsCl, 2mmol/L $MgCl_2$, 10mmol/L HEPES, and 10mmol/L glucose (pH 7.4 (CsOH)). Pipette solution contained 140mmol/L CsCl, 2mmol/L $MgCl_2$, 10mmol/L HEPES, and 10mmol/L EGTA (pH 7.2 (CsOH)).

Western blotting

HiPSC-CMs were collected and sonicated in RIPA buffer (R0278, Merck KGaA, Darmstadt, Germany) and the protein concentration was detected by BCA Protein Assay Kit (23227, Thermo Fisher Scientific, Waltham, MA, USA). The primary antibodies used in western blotting analysis were as follow: Glyceraldehyde 3-phosphate dehydrogenase (GAPDH) (14C10) antibody (5G4, 1:100000; HyTest Ltd, Turku, Finland); anti-Na_v1.5 (SCN5A)(493-511) antibody (ASC-005, 1:500; Alomone Labs, Israel); anti-Ca_v1.2 (CACNA1C) antibody (ASC003, 1:500; Alomone Labs, Israel); anti-NCX1 antibody (ab135735, 1:500; Abcam, Cambridge, UK); anti-K_v4.3 (KCND3) antibody (APC-017, 1:500; Alomone Labs, Israel); anti-hK_v11.1 antibody (P9497, 1:400; Sigma-Aldrich, Merck KGaA, Darmstadt, Germany); anti-KCNQ1 antibody (APC-022, 1:500; Alomone Labs, Israel); anti-Kir2.1/BIK antibody (ab109750, 1:1000; Abcam, Cambridge, UK); DNMT1 antibody (NB100-56519, 1:5000; Novus Biologicals, USA); DNMT2 antibody (19221-1-AP, 1:1000; ProteinTech, IL, USA); DNMT3A antibody (NB120-13888, 1:2000; Novus Biologicals, USA); DNMT3B antibody (NB300-516, 1:500; Novus Biologicals, USA); anti-TET1 antibody-C-terminal (ab101698, 1:1000; Abcam, Cambridge, UK). The secondary antibodies were

anti-rabbit IgG-peroxidase produced in goat (A0545, 1:2000; Sigma-Aldrich, Merck KGaA, Darmstadt, Germany) or anti-mouse IgG-peroxidase produced in goat (A3682, 1:2000; Sigma-Aldrich, Merck KGaA, Darmstadt, Germany).

Immunofluorescence

Cardiomyocytes were dissociated from 24-well plates and plated onto the culture slides (354114; Thermo Fisher Scientific, Waltham, MA, USA). After incubated with cancer cell media or cardio culture medium for 48h, all slides were washed by PBS and fixed with 4% paraformaldehyde at room temperature for 10min and permeabilized with 0.5% triton for 10min. After blocking with 5% BSA (10270106; Thermo Fisher Scientific, Waltham, MA, USA) for 30min, the slides were incubated with primary antibodies at 4˚C, and then were incubated with the second antibodies anti-mouse IgG (H+L), F(ab')2 Fragment (Alexa Fluor 594 Conjugate, 8890, 1:2000; Cell Signaling, USA) and goat anti-rabbit IgG (H+L) highly cross-adsorbed secondary antibody, Alexa Fluor Plus 488 (A32731, 1:2000; Thermo Fisher Scientific, Waltham, MA, USA). The primary antibodies used on hiPSC-CMs were monoclonal anti-α-actinin (Sarcomeric) (A7811, 1:200; Sigma-Aldrich, Merck KGaA, Darmstadt, Germany); anti-Myl4 antibody (PAD427Hu01, 1:200; Cloud-Clone corp, CCC, USA). 3 random fields per wells (at least 10 cells) were photographed by the fluorescence microscope (Leica DMRE, Leica Mikrosysteme Vertrieb GmbH, Wetzlar, Germany), and the fluorescence density of the positive staining per field was measured by Image J software (Research Services Branch, National Institute of Mental Health, Bethesda, Maryland, USA).

Supplementary Tables

Supplementary table 1. Bisulfite sequencing PCR primers for CpG islands

	Primer 5' to 3'		Products
SCN5A CpG island	Forward	TGTGTTTTTAGGAAAGTGTGGT	364bp
	Reverse	ACTAACATACACACCAACACCC	
KCND3 CpG island	Forward	AGTAGTGGGGATTTATGGGTT	340bp
	Reverse	CAACACCTACTTAAAACCAAACC	
KCNQ1 CpG island	Forward	AGGTATTTGATAGTGGTGGTTT	393bp
	Reverse	TTACTCCTACCCTACCCTCA	

Supplementary table 2. Primer sequences for real-time PCR.

Gene	Gene description	Assay ID
GADPH	Glyceraldehyde-3-phosphate dehydrogenase	Hs99999905_m1
SCN5A	Na$_v$1.5	Hs00165693_m1
SCN10A	Na$_v$1.8	Hs01045137_m1
CACNA1C	Ca$_v$1.2	Hs01062258_m1
SLC8A1	NCX1	Hs00167681_m1
KCND3	K$_v$4.3 (I_{to})	Hs00542597_m1
KCNH2	K$_v$11.1 (hERG /I_{Kr})	Hs04234270_m1
KCNQ1	K$_v$7.1, α-subunit of I_{Ks}	Hs00923522_m1
KCNJ2	Inward rectifier K^+ channel Kir2.1	Hs00265315_m1

Supplementary Figures

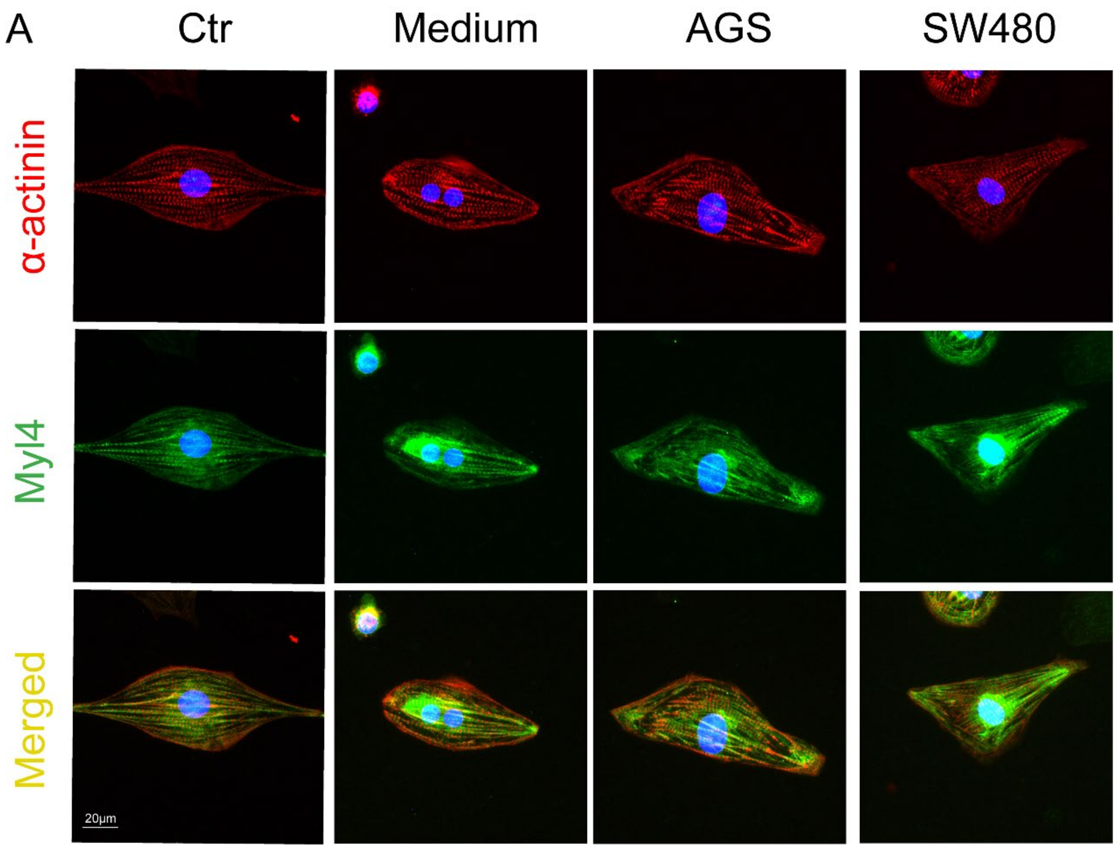

Supplementary figure 1. Immunostaining of hiPSC-CM markers of different groups. Shown is the expression of α-actinin (red) and Myl4 (green) in hiPSC-CMs. “Ctr” represents data from hiPSC-CMs without medium of cancer cells. “Medium” represents data from hiPSC-CMs with addition of fresh medium for cancer cells. “AGS” represents data from hiPSC-CMs with addition of cultured medium of AGS cancer cells. “SW480” represents data from hiPSC-CMs with addition of cultured medium of SW480 cancer cells. Scale bars: 20 μm.

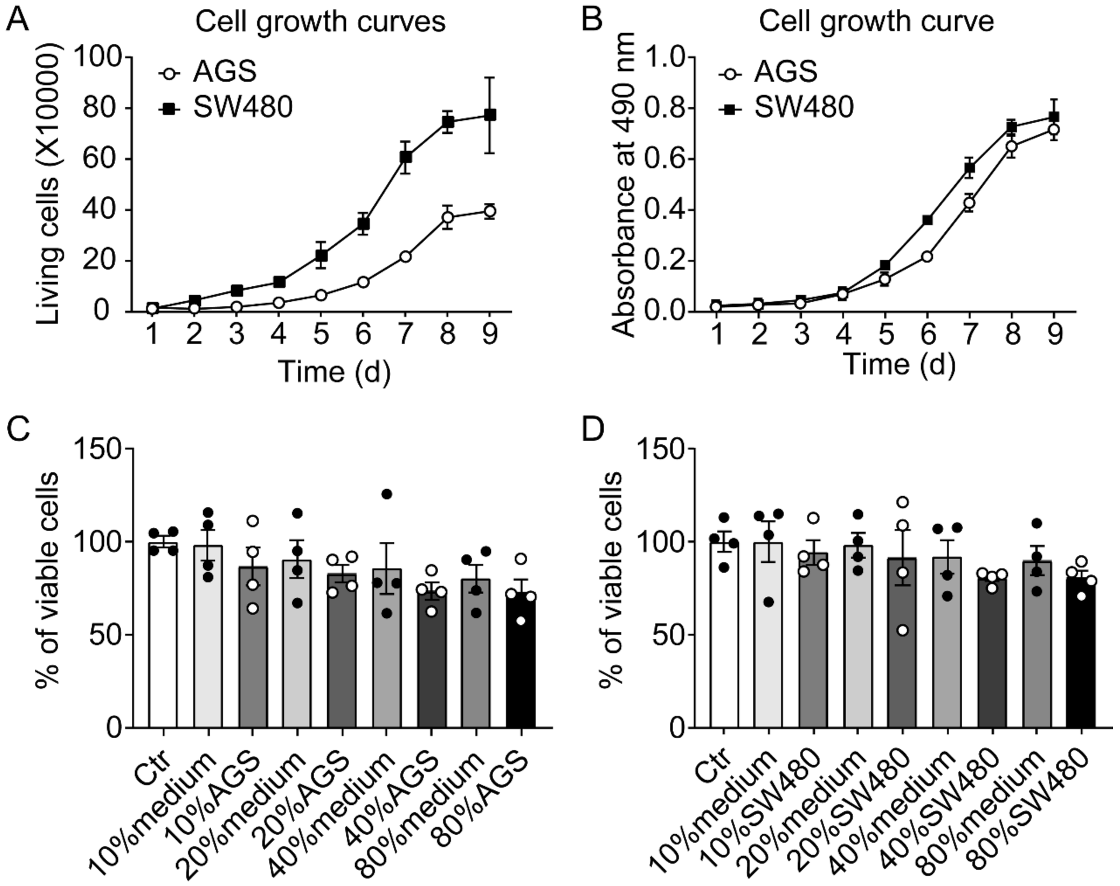

Supplementary figure 2. Cancer cell medium had no effect on cell growth of hiPSC-CMs. (A) Cell growth curve by cell counting. (B) Cell growth curve by MTT assay. (C, D) Cell viability of hiPSC-CMs under different concentrations of supernatant of AGS and SW480 cell culture and medium without cancer cells. “Ctr” represents data from hiPSC-CMs without medium of cancer cells. “Medium” represents data from hiPSC-CMs with addition of fresh medium for cancer cells. “AGS” represents data from hiPSC-CMs with addition of cultured medium of AGS cancer cells. “SW480” represents data from hiPSC-CMs with addition of cultured medium of SW480 cancer cells. Data are presented as mean ± SEM and analyzed by one-way ANOVA. Experiment numbers: n=4.

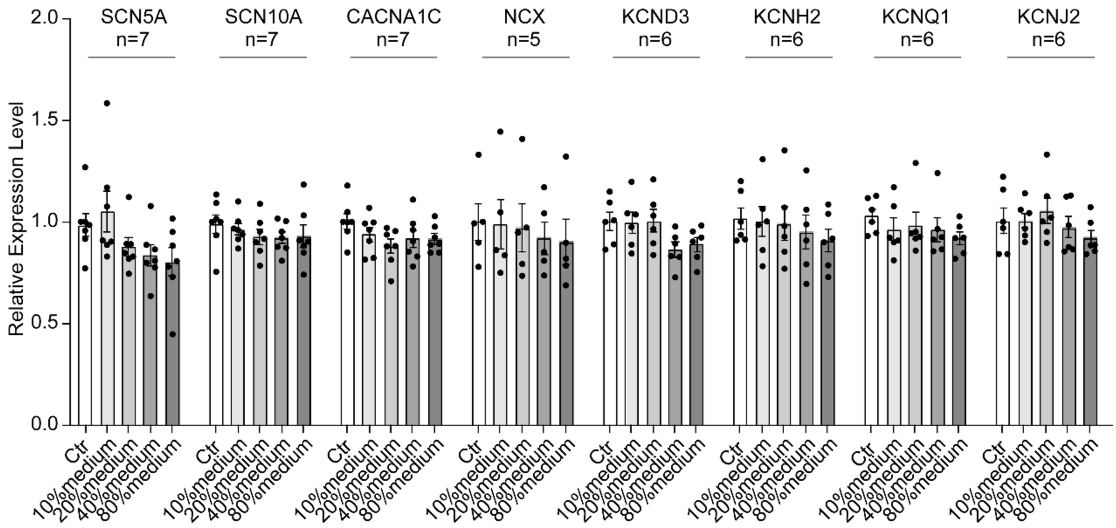

Supplementary figure 3. Ion channel expression profile at mRNA level in hiPSC-CMs cultured with different concentration of fresh cancer culture medium without influence of cancer cells. Different concentration of fresh cancer culture medium from flask without cancer cells were added into flask with hiPSC-CMs for 48h. qPCR analysis was performed to evaluate expression levels of ion channels. “Ctr” represents data from hiPSC-CMs without medium of cancer cells. “Medium” represents data from hiPSC-CMs with addition of fresh medium for cancer cells. Data are presented as mean ± SEM and analyzed by one-way ANOVA. Experiment numbers are indicated as “n”.

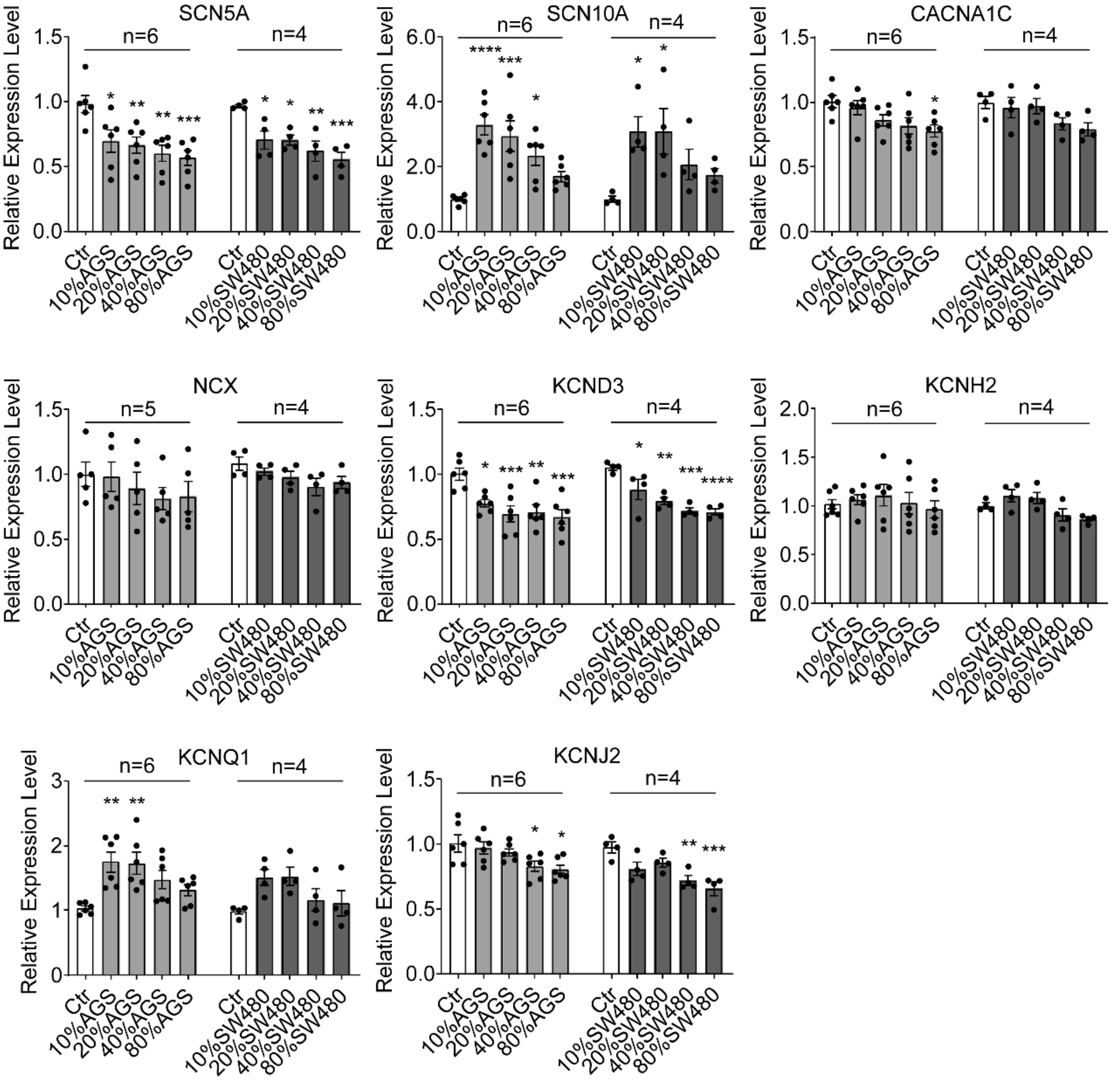

Supplementary figure 4. Ion channel expression profile at mRNA level in hiPSC-CMs cultured with different concentration of AGS and SW480 cancer cell media. Different concentration of cancer culture medium from flask with cancer cells cultured for 8 days were added into flask with hiPSC-CMs for 2 days. qPCR analysis was performed to evaluate expression levels of different ion channels. “Ctr” represents data from hiPSC-CMs without medium of cancer cells. “AGS” represents data from hiPSC-CMs with addition of cultured medium of AGS cancer cells. “SW480” represents data from hiPSC-CMs with addition of cultured medium of SW480 cancer cells. Data are presented as mean ± SEM and analyzed by one-way ANOVA. Experiment numbers are indicated as “n”. **P*<0.05, ***P*<0.01, ****P*<0.001, *****P*<0.0001.

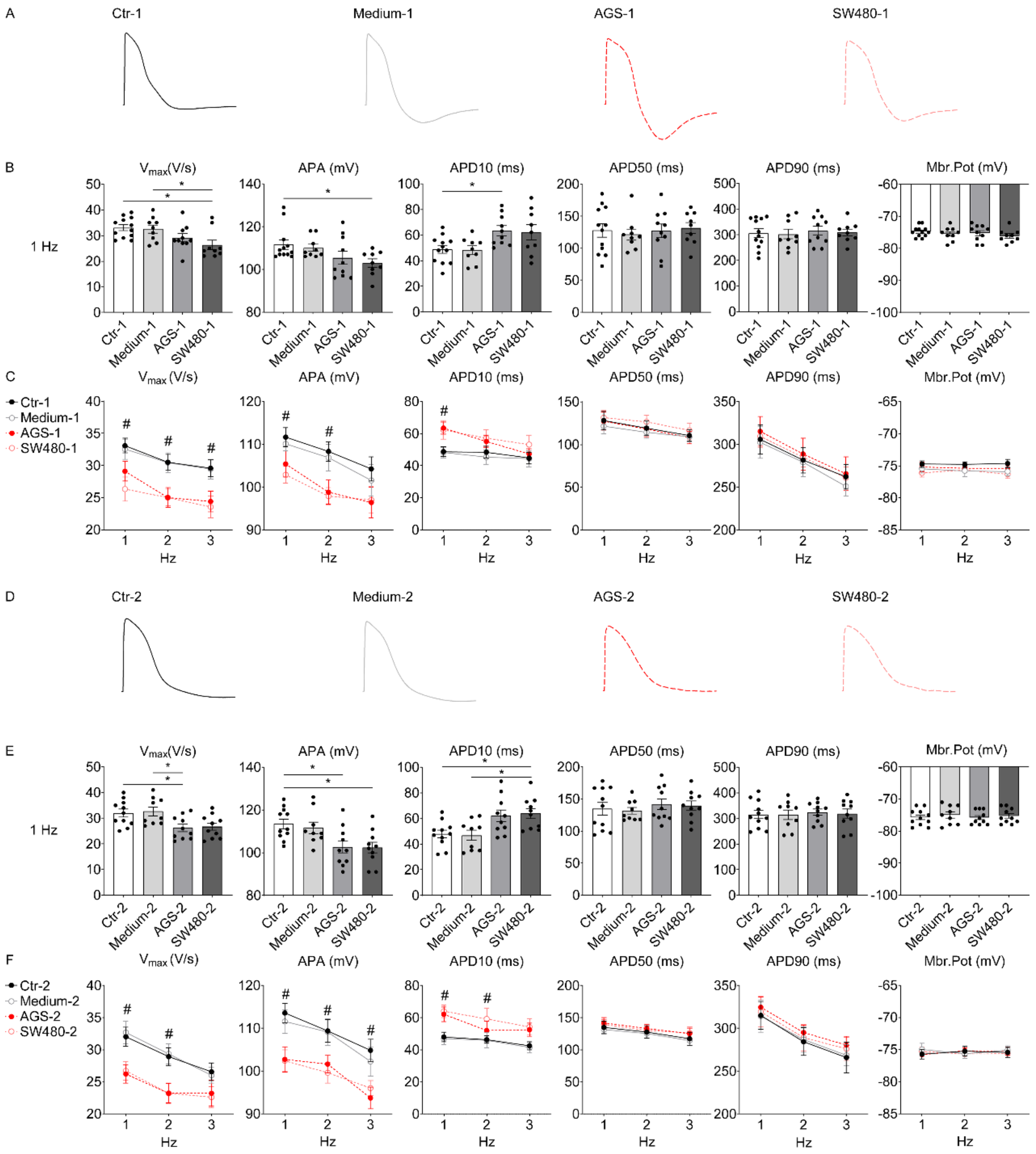

Supplementary figure 5. Action potential (AP) characteristics of hiPSC-CMs cultured with AGS and SW480 cancer cell media in iPSCs from other two donors. APs were recorded by patch clamp (whole cell configuration) measurements in hiPSC-CMs from two donors (represented as "1" and "2") pretreated with culture medium from flask containing cancer cells or no cancer cells. (A, D) Examples of original AP traces in each group. (B, E) Mean values of AP parameters including the AP amplitude (APA), maximal velocity of depolarization (V_{max}), AP duration at 10% (APD10), 50% (APD50) and 90% (APD90) repolarization and resting potential (RP). (C, F) Mean values of AP

parameters at 1, 2 and 3Hz. “Ctr-1, Ctr-2” represents data from hiPSC-CMs without medium of cancer cells. “Medium-1, Medium-2” represents data from hiPSC-CMs with addition of fresh medium for cancer cells. “AGS-1, AGS-2” represents data from hiPSC-CMs with addition of cultured medium of AGS cancer cells. “SW480-1, SW480-2” represents data from hiPSC-CMs with addition of cultured medium of SW480 cancer cells. Data are presented as mean ± SEM and analyzed by one-way ANOVA. (A, B, C) Cell numbers: n=12 in Ctr-1, n=9 in Medium-1, n=10 in AGS-1, n=9 in SW480-1. (D, E, F) Cell numbers: n=11 in Ctr-2, n=9 in Medium-2, n=10 in AGS-2, n=10 in SW480-2. **P*<0.05, ***P*<0.01, ****P*<0.001. (C) # indicates significant differences in V_{max} (Ctr-1 vs SW480-1, Medium-1 vs SW480-1 in 1Hz; Ctr-1 vs AGS-1, Ctr-1 vs SW480-1 in 2Hz; Ctr-1 vs SW480-1 in 3Hz), APA (Ctr-1 vs SW480-1 in 1Hz; Ctr-1 vs AGS-1, Ctr-1 vs SW480-1 in 2Hz), APD10 (Ctr-1 vs AGS-1 in 1Hz); (F) # indicates significant differences in V_{max} (Ctr-2 vs AGS-2, Medium-2 vs AGS-2 in 1Hz; Ctr-2 vs AGS-2, Medium-2 vs AGS-2, Ctr-2 vs SW480-2, Medium-2 vs SW480-2 in 2Hz), APA (Ctr-2 vs AGS-2, Ctr-2 vs SW480-2 in 1Hz; Ctr-2 vs SW480-2 in 2Hz; Ctr-2 vs AGS-2 in 3Hz), APD10 (Ctr-2 vs SW480-2, Medium-2 vs SW480-2 in 1Hz; Ctr-2 vs SW480-2, Medium-2 vs SW480-2 in 2Hz).

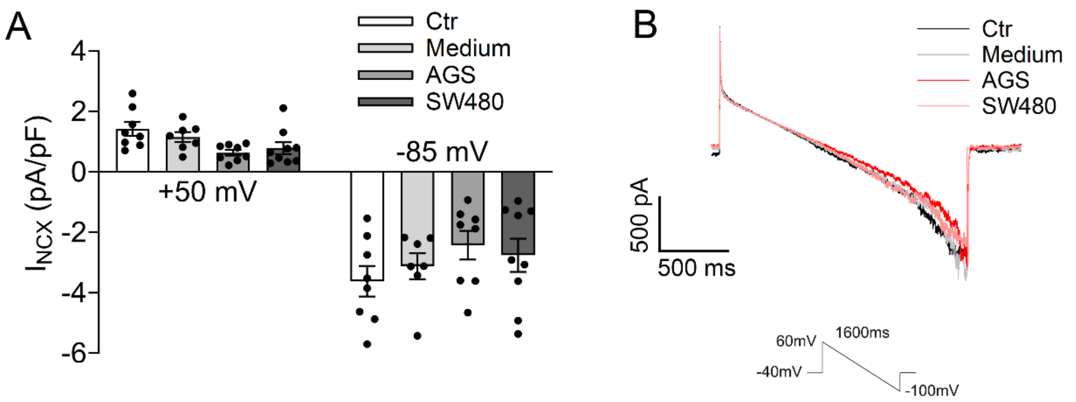

Supplementary figure 6. The sodium-calcium exchanger current (I_{NCX}) in hiPSC-CMs cultured with AGS and SW480 cancer cell media. (A) Mean values of I_{NCX} at 50mV and -85mV. (B) Representative traces of I_{NCX} in Ctr, medium, AGS, and SW480 groups. “Ctr” represents data from hiPSC-CMs without medium of cancer cells. “Medium” represents data from hiPSC-CMs with addition of fresh medium for cancer cells. “AGS” represents data from hiPSC-CMs with addition of cultured medium of AGS cancer cells. “SW480” represents data from hiPSC-CMs with addition of cultured medium of SW480 cancer cells. Data are presented as mean ± SEM and analyzed by one-way ANOVA. Cell numbers: I_{NCX}: n=8 in Ctr, n=7 in Medium, n=8 in AGS, n=9 in SW480.

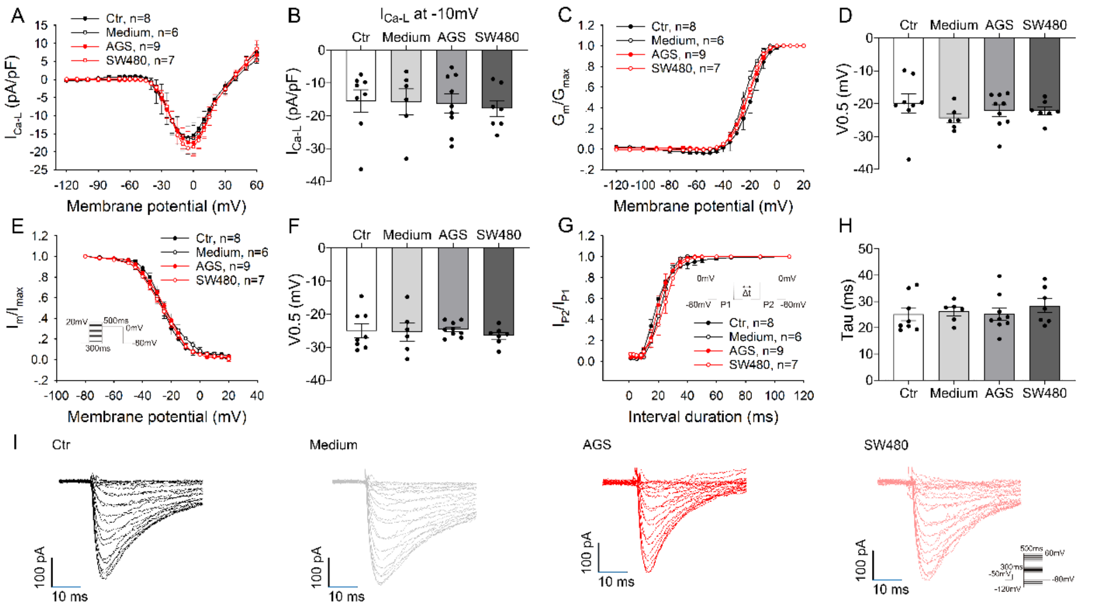

Supplementary figure 7. The L-type calcium current (I_{Ca-L}) in hiPSC-CMs cultured with AGS and SW480 cancer cell media. (A) I-V curves of I_{Ca-L}. (B) Mean values of peak I_{Ca-L} at -10mV. (C) Activation curves of I_{Ca-L}. (D) Mean values of $V_{0.5}$ (the voltage at half maximum) of activation. (E) Inactivation curves of peak I_{Ca-L}. (F) Mean values of $V_{0.5}$ of inactivation. (G) Time course curves of recovery from inactivation of I_{Ca-L}. (H) Mean values of time constants (tau) of recovery from inactivation. (I) Representative traces of I_{Ca-L} in Ctr, medium, AGS, and SW480 groups. “Ctr” represents data from hiPSC-CMs without medium of cancer cells. “Medium” represents data from hiPSC-CMs with addition of fresh medium for cancer cells. “AGS” represents data from hiPSC-CMs with addition of cultured medium of AGS cancer cells. “SW480” represents data from hiPSC-CMs with addition of cultured medium of SW480 cancer cells. Data are presented as mean ± SEM and analyzed by one-way ANOVA. Cell numbers: I_{Ca-L}: n=8 in Ctr, n=6 in Medium, n=9 in AGS, n=7 in SW480.

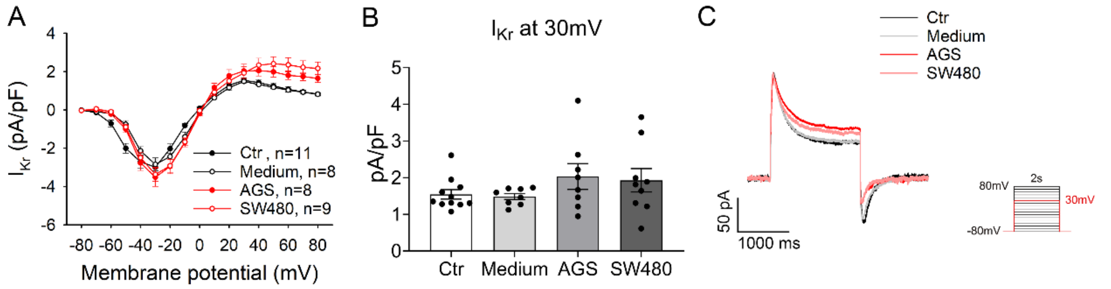

Supplementary figure 8. The rapidly activating delayed rectifier potassium current (I_{Kr}) in hiPSC-CMs cultured with AGS and SW480 cancer cell media. (A) I-V curves of I_{Kr}. (B) Mean values of I_{Kr} at 30mV. (C) Representative traces of I_{Kr} in Ctr, medium, AGS, and SW480 groups. “Ctr” represents data from hiPSC-CMs without medium of cancer cells. “Medium” represents data from hiPSC-CMs with addition of fresh medium for cancer cells. “AGS” represents data from hiPSC-CMs with addition of cultured medium of AGS cancer cells. “SW480” represents data from hiPSC-CMs with addition of cultured medium of SW480 cancer cells. Data are presented as mean ± SEM and analyzed by one-way ANOVA. Cell numbers: I_{Kr}: n=11 in Ctr, n=8 in Medium, n=8 in AGS, n=9 in SW480.

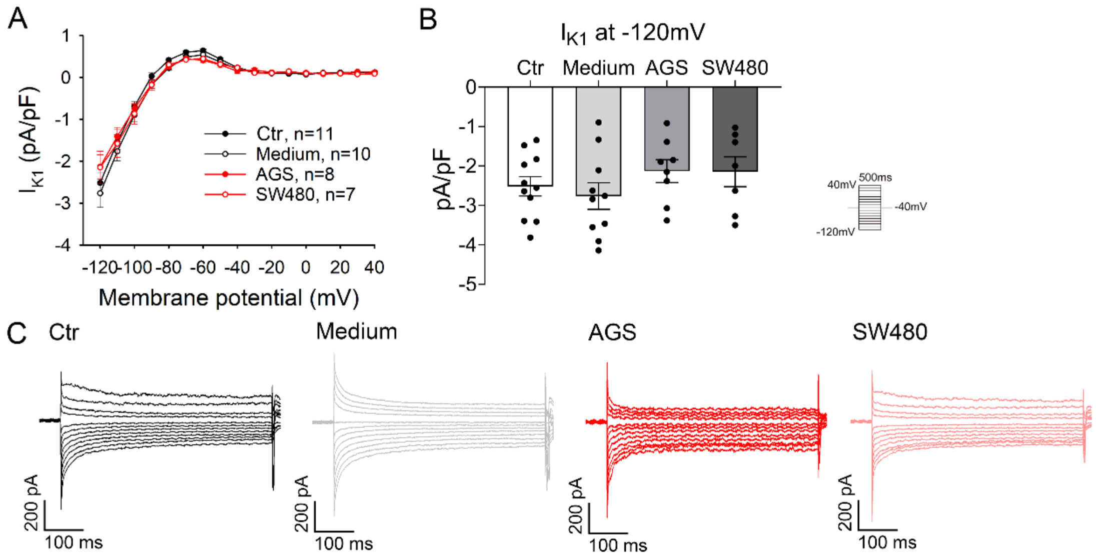

Supplementary figure 9. The inward rectifier potassium current (I_{K1}) in hiPSC-CMs cultured with AGS and SW480 cancer cell media. (A) I-V curves of I_{K1}. (B) Mean values of I_{K1} at -120mV. (C) Representative traces of I_{K1} in Ctr, medium, AGS, and SW480 groups. “Ctr” represents data from hiPSC-CMs without medium of cancer cells. “Medium” represents data from hiPSC-CMs with addition of fresh medium for cancer cells. “AGS” represents data from hiPSC-CMs with addition of cultured medium of AGS cancer cells. “SW480” represents data from hiPSC-CMs with addition of cultured medium of SW480 cancer cells. Data are presented as mean ± SEM and analyzed by one-way ANOVA. Cell numbers: I_{K1}: n=11 in Ctr, n=10 in Medium, n=8 in AGS, n=7 in SW480.

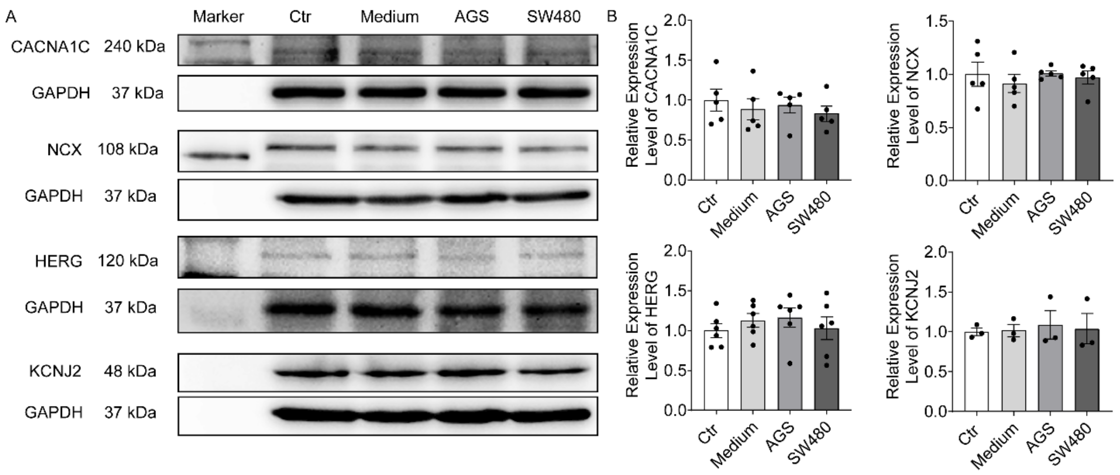

Supplementary figure 10. Protein expression levels of CACNA1C, NCX, HERG, and KCNJ2 in hiPSC-CMs cultured with AGS and SW480 cancer cell media. (A) Protein bands of CACNA1C, NCX, HERG, and KCNJ2. (B) Statistical analysis of relative expression level of CACNA1C, NCX, HERG, and KCNJ2. “Ctr” represents data from hiPSC-CMs without medium of cancer cells. “Medium” represents data from hiPSC-CMs with addition of fresh medium for cancer cells. “AGS” represents data from hiPSC-CMs with addition of cultured medium of AGS cancer cells. “SW480” represents data from hiPSC-CMs with addition of cultured medium of SW480 cancer cells. Data are presented as mean ± SEM and analyzed by one-way ANOVA. Experiment numbers: CACNA1C: n=5, NCX: n=7, HERG: n=6, KCNJ2: n=3.

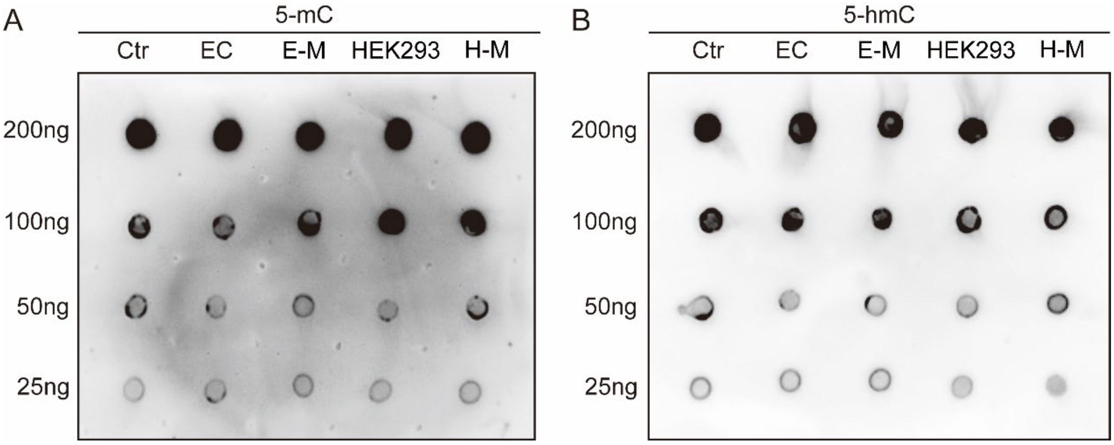

Supplementary figure 11. The whole genome DNA methylation level of hiPSC-CMs after treatment with endothelial cells and HEK293 cells. (A) Whole genome DNA methylation level detected by 5-mC antibody. (B) Whole genome DNA demethylation level detected by 5hmC antibody. “Ctr” represents data from hiPSC-CMs without medium of cancer cells. “EC” represents data from hiPSC-CMs with addition of cultured medium of endothelial cells. “E-M” represents data from hiPSC-CMs with addition of fresh medium for endothelial cells. “HEK293” represents data from hiPSC-CMs with addition of cultured medium of HEK293 cells. “H-M” represents data from hiPSC-CMs with addition of fresh medium for HEK293 cells. Experiment numbers: (A) showed representative image of dot blotting, n=3.

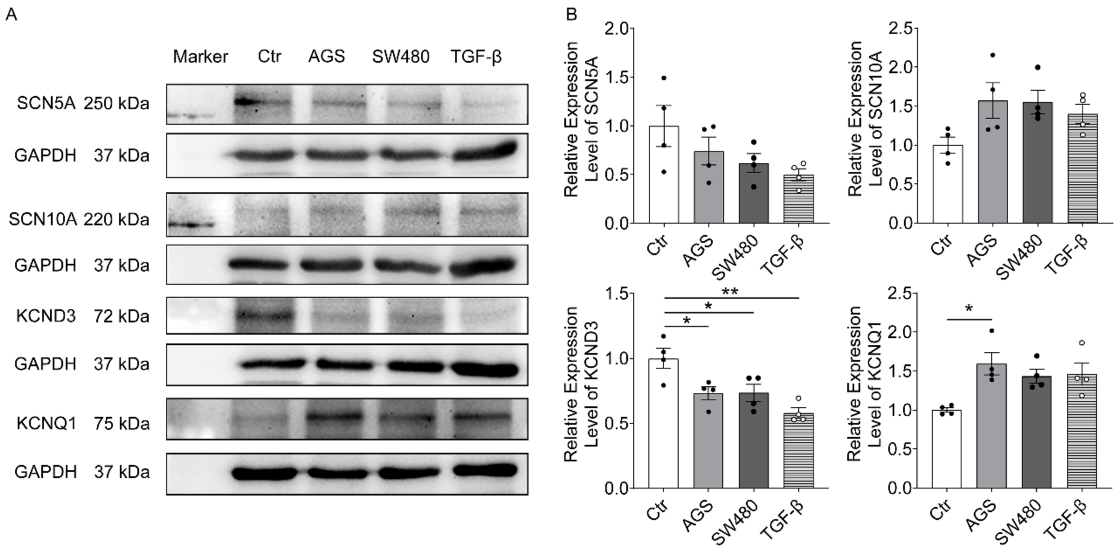

Supplementary figure 12. Protein levels of SCN5A, SCN10A, KCND3, and KCNQ1 after treatment with TGF-β. (A) Protein bands of SCN5A, SCN10A, KCND3, and KCNQ1 after treatment with TGF-β. (B) Statistical analysis of relative expression level of SCN5A, SCN10A, KCND3, and KCNQ1. “Ctr” represents data from hiPSC-CMs without medium of cancer cells. “AGS” represents data from hiPSC-CMs with addition of cultured medium of AGS cancer cells. “SW480” represents data from hiPSC-CMs with addition of cultured medium of SW480 cancer cells. “TGF-β” represents data from hiPSC-CMs with addition of TGF-β. Data are presented as mean ± SEM and analyzed by one-way ANOVA. Experiment numbers: SCN5A: n=4, SCN10A: n=4, KCND3: n=4, KCNQ1: n=4. *$P<0.05$, **$P<0.01$.
